# Safety and feasibility evaluation of tourniquets for total knee replacement (SAFE-TKR): study protocol

**DOI:** 10.1136/bmjopen-2018-022067

**Published:** 2018-04-10

**Authors:** Peter DH Wall, Imran Ahmed, Andrew Metcalfe, Andrew J Price, Kate Seers, Charles E Hutchinson, Helen Parsons, Jane Warwick, Bushra Rahman, Jaclyn Brown, Martin Underwood

**Affiliations:** 1 Warwick Clinical Trials Unit, University of Warwick, Coventry, UK; 2 Trauma and Orthopaedics, University Hospitals Coventry and Warwickshire, Coventry, UK; 3 Nuffield Department of Orthopaedics, Rheumatology and Musculoskeletal Sciences, University of Oxford, Oxford, UK; 4 Royal College of Nursing Research Institute, University of Warwick, Coventry, UK; 5 Warwick Medical School, University of Warwick, Coventry, UK

**Keywords:** knee, stroke, adult surgery

## Abstract

**Introduction:**

This study is designed to determine whether a full randomised controlled trial (RCT) examining the clinical effectiveness and safety of total knee replacement surgery with or without a tourniquet is warranted and feasible.

**Method and analysis:**

Single centre, patient-blinded and assessor-blinded RCT. A computer-generated randomisation service will allocate 50 participants into one of two trial treatments, surgery with or without a tourniquet. The primary objective is to estimate recruitment, crossovers and follow-up of patients. All patients will have an MRI scan of their brain preoperatively and day 1 or 2 postoperatively to identify ischaemic cerebral emboli (primary clinical outcome). Oxford Cognitive Screen, Montreal Cognitive Assessment and Mini-Mental State Examination will be evaluated as outcome tools for measuring cognitive impairment at days 1, 2 and 7 postoperatively. Thigh pain, blood transfusion requirements, venous thromboembolism, revision surgery, surgical complications, mortality and Oxford knee and five-level EuroQol-5D scores will be collected over 12 months. *Integrated qualitative research study*: 30 trial patients and 20 knee surgeons will take part in semistructured interviews. Interviews will capture views regarding the pilot trial and explore barriers and potential solutions to a full trial. *Multicentre cohort study*: UK National Joint Registry data will be linked to Hospital Episode Statistics to estimate the relationship between tourniquet use and venous thromboembolic event, length of hospital stay, risk of revision surgery and death. The study will conclude with a multidisciplinary workshop to reach a consensus on whether a full trial is warranted and feasible.

**Ethics and dissemination:**

National Research Ethics Committee (West Midlands-Edgbaston) approved this study on 27 January 2016 (15/WM/0455). The study is sponsored by University of Warwick and University Hospitals Coventry and Warwickshire. The results will be disseminated via high-impact peer-reviewed publication.

**Trial registration number:**

ISRCTN20873088; Pre-results.

**Strengths and limitations of this study**

- Comprehensive feasibility research.
- Clearly defined outcome measures.
- Patient blinding.

- Single-centre design with small sample size.
- Absence of blinding among clinicians delivering the intervention.

## Introduction

Arthritis of the knee can cause pain and restrict activities of daily living. Total knee replacement (TKR) surgery is a surgical procedure aimed at resolving the symptoms of end-stage knee arthritis.^1^ TKR surgery is typically undertaken with the aid of a tourniquet. A tourniquet acts as an occlusive device around the thigh with the aim of reducing blood flow distally. In the UK, over 90% of TKRs are performed with a tourniquet.^2 3^ Anecdotally, surgeons believe using a tourniquet provides a bloodless field to improve the operative field of view.^4^ Many surgeons also believe using a tourniquet improves the quality of the cementation of the knee implants^5^ by reducing bone bleeding and allowing better interdigitation of the cement into the porous bone.

Previous systematic reviews have concluded that the use of tourniquets did not reduce intraoperative or postoperative blood loss^6^ and were associated with significant complications including venous thromboembolic events (VTEs),^5^ wound infection, bruising and nerve palsy.^5 7^

In TKR surgery, a tourniquet causes both arterial and venous stasis for the duration it is applied. It is therefore unsurprising that the use of a thigh tourniquet might increase the risk of postoperative VTE.^2^ However, VTE may not be the only thromboembolic risk associated with using a tourniquet. Research has demonstrated that systemic emboli can occur when the tourniquet is deflated with up to a 60% prevalence of echogenic material in the circle of Willis.^8 9^ Emboli may reach the systemic circulation through the pulmonary capillaries or the opening of other pulmonary vessels.^9^ The prevalence of postoperative cognitive impairment after TKR is high with reports in the literature varying from 41%–75% at 7 days to 18%–45% at 3 months, and this may in part be explained by cerebral emboli if they are occurring following the release of a tourniquet.^10^

Although studies have demonstrated that tourniquets do not substantially reduce blood loss and may increase complications, a review of these studies, by Alcelik *et al* 7, identified significant design flaws, including issues with randomisation, blinding and the absence of clearly defined outcome measures. Furthermore, no controlled studies have addressed or quantified one of the most potentially serious risks associated with tourniquet, which are cerebral emboli, and any resultant cognitive impairment.

There may be problems with running a trial that involves recruiting patients who, once it is explained, may not be prepared to accept the potential risks of surgery with a tourniquet. Equally, surgeons may be not willing to change surgical practice for a randomised trial.

We designed a feasibility study, which includes three separate but integrated projects: (A) pilot randomised controlled trial (RCT), (B) integrated qualitative research study and (C) retrospective multicentre cohort study.

The objective of the safety and feasiblity evaluation of tourniquets for total knee replacement surgery (SAFE-TKR) study is to establish whether a full RCT evaluating tourniquets in knee replacement surgery is warranted and feasible.

## Pilot RCT

The primary objective of the pilot trial is to estimate recruitment, crossover and follow-up of patients for a full trial.

### Secondary objectives

- evaluate MRI for detecting postoperative ischaemic cerebral emboli, including an estimate of the size and direction of any effect;
- evaluate tools for detecting postoperative cognitive impairment. These tools include Mini-Mental State Examination (MMSE), Montreal Cognitive Assessment (MoCA) and the Oxford Cognitive Screen (OCS), including an estimate of the size and direction of any effect;
- evaluate other candidate primary/coprimary/secondary outcome measures for assessment within a larger trial including: thigh pain, symptomatic VTE, mortality, revision surgery, blood transfusion requirements, function and health-related quality of life. To include obtaining estimates for the SD of continuous outcome variables and differences in proportions for categorical outcome variables in order to facilitate potential sample size calculations for a full trial;
- test and optimise patient information material and the patient pathway for a full trial.

### Method

The protocol was produced in accordance with the Standard Protocol Items: Recommendations for Interventional Trials guidelines.^11^ The trial will be reported in line with the Consolidated Standards of Reporting Trials (CONSORT) statement.^12^

A single-centre two-arm pilot RCT will be performed. All patients under the care of 13 participating orthopaedic consultants at University Hospital Coventry and Warwickshire National Health Service (NHS) trust are potentially eligible for entry into the trial. The following eligibility criteria will be implemented for patient selection:

#### Inclusion criteria

- undergoing a primary unilateral TKR
- age ≥18 years
- able to give written informed consent and to participate fully in the interventions and follow-up procedures.

#### Exclusion criteria

- Patients for whom MRI is contraindicated due to:
  - non-compliant heart pacemaker or defibrillator
  - non-compliant metallic foreign body, for example, in one or both eyes and aneurysm clips in the brain
  - claustrophobia (eg, difficulty in an elevator or telephone box).
- Patients not suitable for a thigh tourniquet (eg, peripheral vascular disease).
- Previous participation in the SAFE-TKR study.

Patients already scheduled for TKR surgery will be screened based on the eligibility criteria. A trained research associate will contact potentially eligible patients via telephone. Patients who are interested in taking part in the study will then be sent a patient information sheet and consent form and a date arranged to answer any further questions about the study and take consent. Verbal consent will initially be taken, followed by signed written consent via post or in person depending on the patient’s preference (see online supplementary appendix 1 and 2).

#### Randomisation

All patients who consent to the trial will be registered and then undergo a preoperative MRI of their brain. Patients will be allocated 1:1 via Warwick randomisation service (independent of the study team) to either TKR surgery with tourniquet or TKR surgery without tourniquet using minimisation to ensure balance between the treatment arms as regards patients with a history of VTE.^13^ To ensure allocation concealment, following enrolment patient details are entered on a web-based form, and the treatment allocation are generated.

#### Planned intervention

Patients will undergo routine elective primary unilateral TKR (cemented) using the standard technique of the anaesthetist and the operating surgeon. Both groups will have a thigh tourniquet applied to the relevant lower limb. Once the patient is fully anaesthetised, one of the following interventions will be applied:

##### Group A (tourniquet inflated)

The tourniquet will be inflated prior to the surgeon creating a wound, and only deflated once the procedure is deemed completed by the surgeon (at a minimum this will be after all the TKR components have been finally inserted).

##### Group B (tourniquet not inflated)

The tourniquet will not be inflated during the procedure.

In line with National Institute for Health and Care Excellence (NICE) guidance, all patients will receive the following routine chemical and mechanical VTE prophylaxis:

- intermittent pneumatic calf compression until patient’s mobility is no longer significantly reduced
- low molecular weight heparin (or unfractionated heparin for patients with severe renal impairment or established renal failure), started 6–12 hours after surgery and continued for 14 days postoperatively.

#### Clinical outcomes and time points

**Table 1:** Clinical outcomes and time points

Time point	Data collection
Baseline	Demographics, total volume of acute brain lesions, MMSE, MoCA, OCS, OKS, EQ-5D-5L, VAS and haemoglobin level.
Day 1	Total volume of acute brain lesions, MMSE, MoCA, OCS, VAS and haemoglobin level.
Day 2	Total volume of acute brain lesions (if not done on day 1), MMSE, MoCA, OCS, VAS and haemoglobin level (if not done on day 1).
Week 1	MMSE, MoCA, OCS, VAS, OKS, EQ-5D, number of intraoperative/postoperative blood transfusions until discharge and surgical complications.
6 months	OKS, EQ-5D and surgical complications.
12 months	OKS, EQ-5D-5L, number of symptomatic VTEs, surgical complications, revision rate and all cause mortality.

Data will be obtained from next of kin and healthcare records.

EQ-5D-5L, five-level EuroQol-5D; MMSE, Mini-Mental State Examination; MoCA, Montreal Cognitive Assessment; OCS, Oxford Cognitive Screen; OKS, Oxford Knee Score; VAS, visual analogue scale; VTEs, venous thromboembolic events.

##### Primary clinical outcome

The primary clinical outcome will be evidence of new acute ischaemic brain lesions on MRI. The total number and volume of acute brain lesions detected on MRI per patient will be recorded. Presurgery MRIs will be obtained no more than 60 days before surgery, and postsurgery MRIs will be obtained up to 2 days after surgery. Diffuse-weighted MRI is the most powerful tool for diagnosing acute ischaemic brain lesion caused by cerebral microembolism providing high level of sensitivity and specificity.^14–16^ The MRIs will all follow a standardised protocol including fast spin-echo fluid-attenuated inversion recovery sequences and diffusion-weighted spin echo-echo planar imaging. The diffusion-weighted sequence will consist of an initial T2-weighted acquisition followed by a second acquisition with the application of diffusion-sensitising gradients in the three orthogonal directions. Lesions will also be described according to the vascular territory (anterior, middle, posterior cerebral arteries or vertebrobasilar arteries), side and type (cortical vs subcortical or deep grey matter). A planimetry of each lesion will be performed by using a grid overlay (using Image Processing and Analysis in Java software, National Institutes of Health) and by calculating lesion volume by multiplying the number of involved grids by the slice thickness and slice gap. Attack rate (presence of new lesions/number of patients) will also be calculated. A new lesion will be defined as a focal hyperintense area detected by the fluid-attenuated inversion recovery sequence, corresponding to a restricted diffusion signal in the diffusion-weighted imaging sequence.^17^ Scans will be read and evaluated by two experienced consultant radiologists blinded to the timing of the imaging, allocated intervention and the neurological status of the patient. Even asymptomatic cerebral emboli, which may be detected using this approach, are associated with gradual memory impairment and cognitive decline.^18–20^

##### Secondary outcomes

1. MoCA preoperatively and days 1, 2 and 1 week postoperatively: the MoCA is a brief cognitive screening tool with high sensitivity and specificity for detecting mild cognitive impairment.^21^
2. OCS preoperatively and days 1, 2 and 1 week postoperatively: the OCS has higher sensitivity than the MOCA for measuring cognitive deficits associated with stroke (spatial disorders and apraxia), and it provides response speed as well as accuracy measures, so that indices such as processing speed can be derived.^22^
3. MMSE scores preoperatively and days 1, 2 and 1 week postoperatively: the MMSE is the most commonly used tool for measuring cognitive impairment and has been used extensively to measure disturbances in postoperative cognition.^23–25^
4. Acute thigh pain preoperatively and on days 1, 2 and 1 week postoperatively: the 100 mm visual analogue scale (VAS) with 0 being no pain and 100 mm being the worst pain is a validated patient-reported outcome measure for pain following TKR surgery.^26^
5. Oxford Knee Score (OKS) preoperatively and at 1 week, 6 and 12 months postoperatively: this is a self-administered, validated knee replacement composite outcome measure of knee pain and function consisting of 12 items. The score ranges from 12 to 60, where 12 represents the best outcome and 60 represents the worst outcome.^27^
6. Five-level EuroQol-5D (EQ-5D-5L) scores preoperatively and at 1 week, six and 12 months postoperatively: This is a validated measure of health-related quality of life, consisting of a five-dimension health status classification system and a separate VAS. EQ-5D-5L is primarily designed for self-completion by respondents and suited for use in postal surveys, in clinics and face-to-face interviews. It is cognitively simple, taking only a few minutes to complete.^28 29^
7. Number of symptomatic VTE events up to 12 months postoperatively: symptomatic VTE events will be defined based on NICE guidance: deep vein thrombosis=swollen or painful leg and a positive proximal leg vein ultrasound scan; pulmonary embolism=chest pain, shortness of breath or haemoptysis and positive CT pulmonary angiogram (CTPA) scan or ventilation perfusion single photon emission computed tomography (V/Q SPECT) or planar scan if CTPA not available. Symptomatic VTEs will be captured throughout the postoperative period via hospital records. In addition, patient questionnaires at 6 and 12 months will capture further data.
8. Surgical complications up to 12 months postoperatively: patient questionnaires and healthcare records will collect adverse events (AEs) that are deemed to be as a direct result of surgery. Two blinded researchers will determine whether AEs should be classified as a surgical complication, where there is disagreement, a third researcher will determine the final allocation.
9. Number of intraoperative/postoperative blood transfusions until discharge: data will be obtained from hospital records and recorded as number of units transfused.
10. Change in haemoglobin concentration (Hb g/L): routinely collected haemoglobin concentrations (Hb g/L) measured from a full blood count taken on days 1–3 postoperatively (the sample closest to day 1 will be favoured) will be subtracted from a preoperative (Hb g/L) measured within 3 months (the sample closest to the date of surgery will be favoured).
11. Revision rate of the TKR prosthesis at 12 months: revision of the prosthesis for any reason will be established by patient questionnaires at 12 months and hospital records.
12. All-cause mortality rates at 12 months: all baseline data will be summarised descriptively by intervention group. The flow of patients through the trial will be presented in a CONSORT diagram, and patients withdrawn are summarised by treatment group.

### Assessment and blinding

It will not be possible to blind the clinicians administering the intervention. Patients and research associates collecting outcome measures will be blinded to treatment allocation. Patients with any outstanding postoperative cognitive tests (typically either day 2 or day 7) after discharge from hospital will have these administered by a trained research associate visiting them at home or an agreed location. Patients will receive a follow-up telephone call at 6 weeks to record any VTEs since surgery. OKS, EQ-5D and complications questionnaire will be posted to the patient at 6 and 12 months. We will use techniques common in long-term cohort studies to ensure minimum loss to follow-up, such as collection of next of kin contact addresses and telephone numbers, mobile telephone numbers and email addresses. A maximum of three attempts will be made acquire outcome data at each time point.

### Sample size calculation

As this is a pilot trial and not designed to measure effect, a formal sample size calculation is not required (the statistical analysis will be largely descriptive). We propose seeking to recruit and obtain primary clinical outcome data for 50 patients for descriptive analysis.

### Statistical analysis

Standard descriptive statistics (eg, medians and ranges or means and variances, dependent on the distribution of the outcome) and graphical plots showing correlations will be presented for the primary and secondary outcome measures. Baseline data will be summarised to check comparability between treatment arms and to highlight any characteristic differences between those individuals in the trial, those ineligible and those eligible but withholding consent. Exploratory analyses using regression techniques will be used to assess change in total volume of acute brain lesions in the two treatment groups and investigate the relationship between cognitive test scores and total volume of acute brain lesions. Linear regression will also be used to estimate the proportion of the variation in total volume of acute brain lesions that may be explained by change in cognition score between baseline and first postoperative assessment. To further investigate the extent to which cognition score is a surrogate for total volume of acute brain lesions, we will use logistic regression to predict treatment group from change in the total volume of acute brain lesions. The relationship between cognitive symptoms and the MRI data will also be evaluated using voxel-based lesion-symptom mapping, where MRI intensity changes are associated to cognitive deficits using either classification scores (deficit or not) or continuous measures of cognition.

The routine statistical analysis will mainly be carried out using STATA V.15 (Data Analysis and Statistical Software).

### Data management

After baseline demographic data is collected, a unique trial number will identify patients. All data collected will be entered into a secure trial database held at Warwick Clinical Trials Unit (WCTU). Identifiable patient information will be held in a locked filing cabinet and coded with a patient trial number. The WCTU quality assurance manager will audit trial records in accordance with standard operating procedures. Outcomes will not be analysed until all primary outcome data are collected.

### Trial oversight

The Trial Management Group (TMG), consisting of the staff involved in the day-to-day running of the study, will meet monthly. Significant issues arising from management meetings will be referred to the Trial Steering Committee (TSC) as appropriate. The trial will be guided by a TSC, a group of respected and experienced personnel and trialists as well as lay representatives. The TSC will have an independent chairperson. At least two formal TSC meetings will be held—one before the trial starts and one before recruitment to the trial completes. As this is a small feasibility study, there will not be a data monitoring committee. AEs and serious AEs will be monitored by the investigators. AEs will be assessed for causality within 24 hours of notification and patients followed up as per protocol. The trial may be stopped prematurely if mandated by ethics committee, a major unexpected safety concern arises or funding ceases. Any proposed changes to the protocol will first be reviewed by the TSC and, if approved, it will be submitted to the trial sponsor, funding body and local research ethics committee. All approved protocols will be marked by a version number and date. Requests for access to the final dataset will be overseen by the TSC. Reasonable requests will then be given access to a full anonymised dataset.

### Patient and public involvement (PPI)

The study has a PPI group that includes two patients (CG and JS) who have previously undergone TKR surgery and have experience of the intervention being evaluated and its burden on patients. The study also has one public member (JD). The PPI group helped develop this study protocol, the associated patient information material and the outcome measures to be used through an active participation in both TMG and TSC meetings. The PPI group critically evaluate study progress and are active study collaborators alongside other members of the research team. The PPI group have taken part in training events to help them participate fully as members of the study team. The PPI group will facilitate the preparation of information about the results of this study and any future planned larger scale study to inform participants of this study, pateints and the wider public. This information will be disseminated through a mixture of social media, written information sheets and peer-reviewed published papers.

## Integrated qualitative research study

### Method

#### Patients

In-depth semistructured interviews among with randomised patients and potential patients who decline to take part in the pilot trial will help understand people’s views regarding participation in the pilot trial. A purposive sample of around 30 people (evenly split between recruited and not recruited) will be interviewed, based on patient demographics (including age, gender and socioeconomic status).^30 31^ Patients will be recruited by a trained research associate and interviews will then be undertaken at a time convenient to the patient.

#### Surgeons

A survey will be undertaken among surgeons who routinely do TKR surgery. The survey will help gauge the extent to which this community is in clinical equipoise and would be willing to engage with and support a larger trial. We will use a web-based survey of members of the British Association for Surgery of the Knee (BASK). BASK has a UK membership of over 100 practising knee surgeons of whom the majority routinely undertake TKR surgery. A sample of at least 20 BASK surgeons (both those clearly in equipoise and those that are not) will be invited to take part in more in-depth qualitative interviews. We will use the general theory of implementation to investigate and understand the core constructs of sense-making processes of patients and surgeons regarding the feasibility of a full trial and to help understand any barriers and enablers for a larger trial.

Digital audio recordings of interviews will be transcribed verbatim, checked and anonymised. Data will be managed and shared using NVivo analysis software. The analysis will be informed by a constant comparative approach, where early analysis informs subsequent data collection, and data analysis takes place alongside data collection.^30^ This means insights from patients and surgeons can be explored further in the ongoing interviews. Data are coded from the start of data collection, and new data are compared with existing data. Codes are compared; categories are constructed and explored to ensure they are robust and then they are linked with relevant theoretical literature. A technique of triangulation protocol will be used to facilitate the integration of the findings of the qualitative research with the quantitative data, thereby helping to determine the overall feasibility of a full trial.^32^ The data from patient interviews will also be used to help design optimal patient information materials for a larger trial.

## Multicentre cohort study

The National Joint Registry (NJR) is a population-based registry of joint replacements in the UK and covers both the NHS and private sectors. From April 2003 to December 2003, the NJR collected data on the use of tourniquets for TKR surgery in England and Wales. At this stage, there were 406 hospitals listed within the NJR system (NHS hospitals, independent sector hospitals and treatment centres—both NHS funded and privately funded) and of these, 384 returned data (ie, 94%). The NJR dataset also contains many other key variables that are known to affect mortality, implant survivorship (revision rate) and the risk of VTE (1) use and type of VTE prophylaxis (such as low molecular weight heparin, aspirin and intermittent calf pump), (2) type of implant used, (3) use of cement and (4) basic patient demographics including age, body mass index (BMI) and American Society of Anesthesiologists (ASA) grade.

### Method

We will access NJR data for the period in which tourniquet use was collected as part of the minimum dataset standard for knee replacement surgery: April 2003 to December 2003. We will analyse only data from primary cemented TKRs. Tourniquet use along with other routinely collected NJR baseline variables—age at time of surgery, sex and ASA classification—will be analysed to measure for independent effects on all cause revision. A tourniquet will be classed as used if even for a selective period of the procedure such as cementation. Sociodemographics, comorbidities, VTEs, cerebrovascular accident (CVAs or stroke) and length of hospital stay will be obtained by using patient identifiers (NHS number, gender, date of birth, postcode, procedure code and local patient identifier code) from the NJR to link the data to the Hospital Episode Statistics (HES) database. The HES data will provide details of VTE, sociodemographics, comorbidities and hospital length of stay for the TKR. The study will specifically estimate differences between groups (tourniquet or no tourniquet) for the following:

- baseline characteristics (age, BMI, ASA, comorbidities, type of VTE prophylaxis, type of implant and sociodemographics)
- all-cause mortality up to 12 months
- length of inpatient hospital stay
- risk of surgical complications up to 30 days
- risk of VTE up to 12 months
- risk of CVA up to 12 months
- risk of revision at 1, 5 and 10 years.

### Statistical Analysis

The Kaplan-Meier method will be used to calculate mortality and revision rates in those who underwent TKR with and without a tourniquet. Multivariate logistic regression will be used to assess the effect of age, BMI, socioeconomic status, tourniquet use, type of VTE prophylaxis, ASA grade, comorbidities and type of TKR implant (cemented or uncemented) on risk of VTE, CVA and risk of revision. The results of these analyses will inform the planning of a subsequent definitive full trial, if such a trial is feasible.

## Conclusions: safety and feasibility evaluation and pathway to a full trial

A workshop will be held at the end of the feasibility study, involving key stakeholders (approximately 30 participants), patients, surgeons, researchers, allied healthcare professionals and healthcare policy makers. The workshop will have an independent chairperson. Using consensus conference methodology, delegates will be presented with results from the pilot RCT, retrospective multicentre cohort study, existing published data and where applicable pooled data from all three sources. The purpose will be to agree a consensus statement on the appropriateness and feasibility of proceeding to a full trial and the most appropriate primary outcome measure for a full trial.

A full definitive trial will be deemed feasible if:

- the pilot RCT and integrated qualitative study suggest patients can be recruited and surgeons are prepared to perform surgery with or without a tourniquet;

and

- the pilot trial identifies either a measure of cognitive brain function that accurately reflects symptomatic brain emboli or an alternative primary/coprimary outcome measure is identified such as pain, symptomatic VTE, revision rates OKS or EQ5D-5L scores that can be accurately and robustly collected in a full trial;

and

- key stakeholders (patients, surgeons, public representatives and researchers) agree at an end of study consensus conference that in light of the feasibility study and existing published research there remains insufficient data to make recommendations about the safety and clinical effectiveness of tourniquets and that a full trial is feasible and there is an appropriate primary/coprimary outcome measure for assessment.

10.1136/bmjopen-2018-022067.supp1 Supplementary data

10.1136/bmjopen-2018-022067.supp2 Supplementary data

## Supplementary Material

Reviewer comments

Author's manuscript
