# Supplementary material for: Safety and feasibility evaluation of tourniquets for total knee replacement (SAFE-TKR): study protocol
Source: BMJ Open. 2018 Apr 10;8(4):e022067. doi: 10.1136/bmjopen-2018-022067 (PMC5900804; doi:10.1136/bmjopen-2018-022067)
Supplement: Supplementary data [file bmjopen-2018-022067supp001.pdf]

WARWICK
THE UNIVERSITY OF WARWICK

University Hospitals Coventry and Warwickshire NHS Trust

Safety and Feasibility Evaluation of Tourniquets For Total Knee Replacement (SAFE-TKR) Study

Patient Information Sheet

Pilot Randomised Controlled Trial

Chief Investigator: Peter Wall

You are invited to take part in our research study. Before you decide whether to take part we would like you to understand why the research is being done and what it would involve for you. Once you have had a chance to read and understand this information sheet a member of our team will go through the information with you and answer any questions you may have.

Background

When you have a total knee replacement, most surgeons apply a tourniquet around the thigh before starting the operation. The tourniquet is a device wrapped around your thigh which when inflated squeezes the thigh, to reduce the blood flow, helping to create a bloodless operative field. Excessive blood within the operative field can interfere with visualising the knee joint during surgery and so a tourniquet is often helpful but by no means essential. There is some limited evidence that a bloodless field during surgery helps to improve the fixation of the knee replacement implant to the bone which could help the implant to last longer.

However, there is some evidence that suggests using a tourniquet during knee replacement surgery may have some risks. Potentially, these include an increased risk of developing a blood clot (so called Deep Vein Thrombosis and Pulmonary Embolism) after surgery. There is also a theoretical risk that when the tourniquet is deflated at the end of the operation that small blood clots that have accumulated in the leg could travel to the brain and cause damage. In addition using a tourniquet during surgery may lead to increased swelling and pain after the operation.

The aim of this study is to help identify and assess all these benefits and risks. By monitoring you closely before, during and after surgery and performing detailed brain scans (Magnetic Resonance Imaging - MRI) before and the day after your operation we will try to establish if there is any evidence that using a tourniquet increases your risk of a blood clot traveling to the brain.

It is important to understand that most patients currently have knee replacement surgery performed using a tourniquet and it remains a very safe procedure. This study will however help to make it even safer and determine for future patients the most effective and safe way of performing a knee replacement.

If you have any questions please contact the Study Manager, Ms Bushra Rahman by either telephoning 02476 968626 / 0787 6876 978 or emailing safe-tkr@warwick.ac.uk
For more information about the study visit:
http://www2.warwick.ac.uk/fac/med/research/hscience/ctu/trials/safetkr

Why have you been invited?
We are asking up to 50 patients who are going to have a total knee replacement to take part in the study. However, if you have a pacemaker, metallic clips in your brain or are claustrophobic you are not eligible to take part because the study involves the use of the MRI scanner.

Do I have to take part?
It is up to you to decide. If you decide to take part we will ask you to sign a consent form. You are free to withdraw at any time without giving a reason. This will not affect the care you receive.

What will happen to me if I take part?
If you decide to take part you will be asked to sign a consent form. You will then be invited to one of the two treatments. In order to make our study work it is crucial that we have equal numbers of volunteers in each treatment group and that the one you (are invited to) join is determined by a sophisticated machine designed for this purpose, not influenced by us. More information about the two possible treatments is given below. Whichever treatment you have your care will be based on meeting your individual needs, and you will continue with the same team of surgeons and healthcare professionals. To ensure the study is as robust as possible and prevent any bias we will inform you whether your surgery involved a tourniquet or not at the end of the study period i.e. 12 months from when you joined the study.

Before the Surgery
We will ask you to complete a questionnaire and a trained member of the research team will ask you some questions. We will also arrange for you to have a MRI brain scan before your operation. The scan will take 20 minutes to complete and is non-invasive. The scan does not use radiation; instead it uses a magnetic field to look at structures in the brain. The MRI scan would involve positioning yourself on a bed, whilst going through a "doughnut shaped" scanner (see image below). During the scan you will be asked to keep still. The scanner can be noisy and headphones will be provided. There will be a radiographer assisting and talking to you at all times throughout the process.

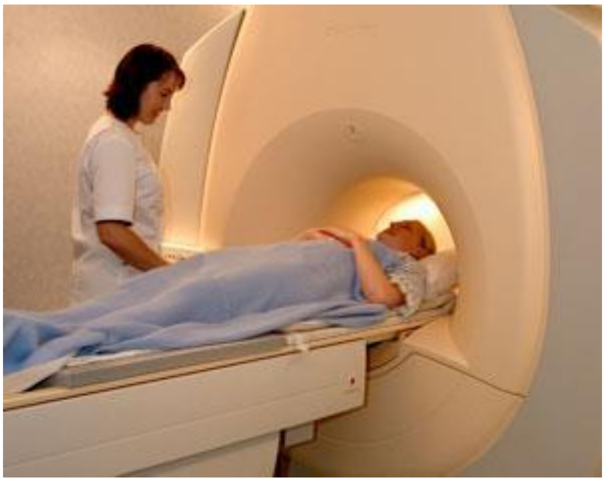
Image of a patient having an MRI Scan

If you have any questions please contact the Study Manager, Ms Bushra Rahman by either telephoning 02476 968626 / 0787 6876 978 or emailing safe-tkr@warwick.ac.uk
For more information about the study visit:
http://www2.warwick.ac.uk/fac/med/research/hscience/ctu/trials/safetkr

After the Surgery
Following the operation you will have another MRI brain scan, before you are discharged from hospital. In the days after your operation you will also be asked to answer some questions to help us test your memory and other brain functions as well as questions about any thigh pain you may be experiencing and how you are feeling in general. These questions will be undertaken by a trained member of the research team at Day 1 and 2 and 7 this may take upto 40 minutes. We will arrange a visit if you are already discharged after your operation. We will call you by telephone 6 weeks after your operation to find out about any problems after the surgery. We will also ask you to complete a postal questionnaire at 6 months and 12 months after your operation to find out how your knee replacement is doing. If you agree we can send you a text message or email near the time to remind you that the questionnaire is due. If you need help completing a questionnaire, a researcher can contact you by phone to help you complete it.

Finally to help understand the reasons why you decided to, or declined to take part in the research we may invite you to undertake a short interview with a trained researcher. This interview will help us to improve the research experience for future patients.

Which treatments are you comparing?
The two treatments that are being compared are:

- *Total knee replacement surgery using a tourniquet*
- *Total knee replacement surgery not using a tourniquet*

What are the possible benefit and risks of taking part?
There are no specific benefits to taking part. Both treatments are designed to help you and your knee arthritis. Your surgeon is able to do the total knee replacement either with or without a tourniquet but it is not clear which way is better. There are some small risks associated with both techniques including blood clots, infection and pain.

What if new information becomes available?
Sometimes during the course of a study, new information becomes available about the treatments that are being studied. If this happens, someone from our research team will tell you about it and discuss with you whether you want to continue in the study. If you decide to withdraw, you can discuss your continued care with your doctor. If you decide to continue in the study you might be asked to sign an updated consent form. Also, on receiving new information, we might consider it to be in your best interests to withdraw you from the study. If this happens we will explain the reasons to you and arrange for your care to continue.

What happens when the research study stops?
You will participate in the study for one year. If you are having any problems relating to the knee surgery after this time, we will arrange for you to see your specialist to continue your care.

What if something goes wrong?
In the event that something goes wrong and you are harmed during the research due to someone's negligence, then you may have grounds for legal action for compensation against the University of Warwick (contact the Head of Research Governance,

Research Impact Services, University House, University of Warwick, Coventry, CV4 8UW or by email researchgovernance@warwick.ac.uk or telephone 02476 522746)

Will my taking part in this study be kept confidential?
All information which is collected about you during the course of the research will be kept strictly confidential. Research data including your name and address will be sent to the University of Warwick so that research staff can stay in touch with you over the course of the year, and send you follow-up questionnaires at 6 and 12 months by post or email. These details will be sent from the hospital by secure means, and kept in locked filing cabinets or in password-protected computer databases accessible only to essential research personnel at the University of Warwick. All other information about you which leaves the hospital will have your name and address removed so that you cannot be recognised from it. If you agree, your GP and other doctors who may treat you, but are not part of this study, will be notified that you are taking part in this study.

What will happen to the results of the research study?
At the end of the study we will publish the findings in medical journals and at medical conferences. You will not be identified in any reports or publications resulting from the study. If you would like to obtain a copy of the published results, please email safe-tkr@warwick.ac.uk.

Who has reviewed this project?
This study has been reviewed and approved by West Midlands – Edgbaston Research Ethics Committee REC Ref. 15/WM/0455. Approval was granted on 27th January 2016

Who is organising and funding the research?
This research has been organised by the University of Warwick and funded by the National Institute for Health Research, UK.

Contacts for further information
If, at any time, you would like further information about this research study you may contact the study manager (Ms Bushra Rahman) by either telephoning 02476 968626 / or emailing safe-tkr@warwick.ac.uk.

For independent advice contact the PALS service (Patient Advice Liaison Service) www.pals.nhs.uk / 0800 0284203.

Thank you for considering participation in this study and for taking time to read this information sheet

If you have any questions please contact the Study Manager, Ms Bushra Rahman by either telephoning 02476 968626 / 0787 6876 978 or emailing safe-tkr@warwick.ac.uk
For more information about the study visit:
http://www2.warwick.ac.uk/fac/med/research/hscience/ctu/trials/safetkr
