# Supplementary material for: Safety and feasibility evaluation of tourniquets for total knee replacement (SAFE-TKR): study protocol
Source: BMJ Open. 2018 Apr 10;8(4):e022067. doi: 10.1136/bmjopen-2018-022067 (PMC5900804; doi:10.1136/bmjopen-2018-022067)
Supplement: Supplementary data [file bmjopen-2018-022067supp002.pdf]

WARWICK
THE UNIVERSITY OF WARWICK

University Hospitals Coventry and Warwickshire NHS Trust

Office Use Only:

Participant ID: ☐☐☐

CONSENT FORM

Pilot Randomised Controlled Trial

SAFE-TKR Study

Chief Investigator: Mr Peter Wall

(Please initial)

1. I confirm that I have read and understand the information sheet dated 09th August 2017 Version 4 for the above study. I have had the opportunity to consider the information, ask questions and have had these answered satisfactorily. ☐

2. I understand that my participation is voluntary and that I am free to withdraw at any time, without giving any reason, without my medical care or legal rights being affected. ☐

3. I understand that relevant sections of any of my medical notes and data collected during the study may be looked at by responsible individuals from the University of Warwick, from regulatory authorities, or from the NHS trust, where it is relevant to my taking part in this research. I give permission for these individuals to have access to my records. ☐

4. I agree to my GP being informed of my participation ☐

5. I agree to being sent a text message reminding that my follow up questionnaire is due. ☐

6. I agree to take part in the above study. ☐

Name of Patient ____________ Signature ____________ Date ____________

Name of Person taking consent ____________ Signature ____________ Date ____________

FOR OFFICE USE ONLY

Copies: ☐ 1 for Patient ☐ 1 for Hospital notes ☐ Original document retained in site file

Signature ____________ Date ____________
