# Supplementary material for: Safety and feasibility evaluation of tourniquets for total knee replacement (SAFE-TKR): study protocol
Source: BMJ Open. 2018 Apr 10;8(4):e022067. doi: 10.1136/bmjopen-2018-022067 (PMC5900804; doi:10.1136/bmjopen-2018-022067)
Supplement: Author's manuscript [file bmjopen-2018-022067.draft_revisions.pdf]

BMJ Open

BMJ Open is committed to open peer review. As part of this commitment we make the peer review history of every article we publish publicly available.

When an article is published we post the peer reviewers' comments and the authors' responses online. We also post the versions of the paper that were used during peer review. These are the versions that the peer review comments apply to.

The versions of the paper that follow are the versions that were submitted during the peer review process. They are not the versions of record or the final published versions. They should not be cited or distributed as the published version of this manuscript.

BMJ Open is an open access journal and the full, final, typeset and author-corrected version of record of the manuscript is available on our site with no access controls, subscription charges or pay-per-view fees (http://bmjopen.bmj.com).

If you have any questions on BMJ Open's open peer review process please email info.bmjopen@bmj.com

BMJ Open

Safety and feasibility evaluation of tourniquets for total knee replacement (SAFE- TKR): study protocol

Journal:	*BMJ Open*
Manuscript ID	bmjopen-2018-022067
Article Type:	Protocol
Date Submitted by the Author:	07-Feb-2018
Complete List of Authors:	Wall, Peter ; Warwick Clinical Trials Unit, Warwick Medical School<br>Ahmed, Imran; University Hospital Coventry<br>Metcalfe, Andrew; University of Warwick, Clinical Trials Unit, Warwick Medical School<br>Price, Andrew; University of Oxford, Nuffield Department of Orthopaedics, Rheumatology and Musculoskeletal Sciences<br>Seers, Kate; Royal College of Nursing Research Institute, Warwick Medical School, University of Warwick, RCN RI<br>Hutchinson, Charles; University of Warwick, Warwick Medical School<br>Parsons, Helen; University of Warwick, Division of Health Sciences<br>Warwick, Jane; University of Warwick Warwick Medical School, Warwick Clinical Trials Unit<br>Rahman, Bushra; University of Warwick, Clinical Trials Unit<br>Brown, Jaclyn; University of Warwick, Clinical Trials Unit<br>Underwood, Martin; Warwick University, Warwick Medical School<br>SAFE-TKR study group , On behalf of; University of Warwick, Clinical Trials Unit
Keywords:	Adult orthopaedics < ORTHOPAEDIC & TRAUMA SURGERY, Knee < ORTHOPAEDIC & TRAUMA SURGERY, Orthopaedic & trauma surgery < SURGERY

SCHOLARONE™
Manuscripts

Safety and feasibility evaluation of tourniquets for total knee replacement (SAFE- TKR): study protocol

Authors
Peter DH Wall, Imran Ahmed, Andrew Metcalfe, Andrew Price, Kate Seers, Charles E Hutchinson, Helen Parsons, Jane Warwick, Bushra Rahman, Jaclyn Brown, Martin Underwood on behalf of the SAFE-TKR Study Group.

Corresponding author
Mr Peter David Henry Wall
Clinical Lecturer in Trauma and Orthopaedic Surgery
Warwick Clinical Trials Unit
Warwick Medical School
University of Warwick
Coventry
CV4 7AL
p.d.h.wall@warwick.ac.uk

Peter Wall: Warwick Clinical Trials Unit, University of Warwick, Coventry, UK, CV4 7AL
Imran Ahmed: University Hospital Coventry Hospital, Clifford Bridge Road, Coventry, UK, CV2 2DX.
Andrew Metcalfe: Warwick Clinical Trials Unit, University of Warwick, Coventry, UK, CV4 7AL
Andrew Price: Nuffield Department of Orthopaedics, Rheumatology and Musculoskeletal sciences, Oxford, UK
Kate Seers: Royal College of Nursing Research Institute, University of Warwick, Coventry, UK, CV4 7AL
Charles Hutchinson: Warwick Medical School, University of Warwick Coventry, UK, CV2 2DX.
Helen Parsons: Warwick Clinical Trials Unit, University of Warwick, Coventry, UK, CV4 7AL
Jane Warwick: Warwick Clinical Trials Unit, University of Warwick, Coventry, UK, CV4 7AL
Bushra Rahman: Warwick Clinical Trials Unit, University of Warwick, Coventry, UK, CV4 7AL
Jaclyn Brown: Warwick Clinical Trials Unit, University of Warwick, Coventry, UK, CV4 7AL
Martin Underwood: Warwick Clinical Trials Unit, University of Warwick, Coventry, UK, CV4 7AL
SAFE-TKR Study group: Warwick Clinical Trials Unit, University of Warwick, Coventry, UK, CV4 7AL

Word count: 4077

For peer review only

Abstract for protocol

Introduction
This study is designed to determine whether a full randomised controlled trial (RCT) examining the clinical effectiveness and safety of total knee replacement (TKR) surgery with or without a tourniquet is warranted and feasible.

Method and analysis
Single centre, patient and assessor blinded RCT. A computer generated randomisation service will allocate 50 participants into one of two trial treatments, surgery with or without a tourniquet. The primary objective is to estimate recruitment, crossovers and followup of patients. All patients will have a Magnetic Resonance Imaging (MRI) scan of their brain preoperatively and day 1 or 2 postoperatively to identify ischaemic cerebral emboli (primary clinical outcome). Oxford Cognitive Screen, Montreal Cognitive Assessment, Mini-Mental State Examination will be evaluated as outcome tools for measuring cognitive impairment at day 1, 2 and 7 postoperatively. Thigh pain, blood transfusion requirements, venous thromboembolism, revision surgery, surgical complications, mortality and Oxford knee and EQ-5D-5L Scores will be collected over 12 months.
Integrated qualitative research study: 30 trial patients and 20 knee surgeons will take part in semi-structured interviews. Interviews will capture views regarding the pilot trial and explore barriers and potential solutions to a full trial.
Multi-centre cohort study: UK National Joint Registry (NJR) data will be linked to Hospital Episode Statistics (HES) to estimate the relationship between tourniquet use and VTE, length of hospital stay, risk of revision surgery and death.
The study will conclude with a multidisciplinary workshop to reach a consensus on whether a full trial is warranted and feasible.

Ethics and dissemination
National Research Ethics Committee (West Midlands-Edgbaston) approved this study on 27/01/2016 (15/WM/0455). The study is sponsored by University of Warwick and University Hospitals Coventry and Warwickshire. The results will be disseminated via high impact peer-reviewed publication.
Funding: National Institute for Health Research Post-Doctoral Fellowship Award (NIHR-PDF-2015-08-108).
Trial Registration: ISRCTN20873088

Article Summary
Strengths and limitations of the study
Strengths

- Comprehensive feasibility research
- Clearly defined outcome measures
- Patient blinding

Limitations

- Single centre design with small sample size
- Absence of blinding among clinicians delivering the intervention

For peer review only

Introduction

Arthritis of the knee, which affects approximately 1 in 2 people before the age of 85[1], can cause pain and restrict activities of daily living. Total knee replacement (TKR) surgery is a surgical procedure aimed at resolving the symptoms of end stage knee arthritis[2].

Many lower limb orthopaedic procedures, including TKR surgery, are undertaken with the aid of a tourniquet. A tourniquet acts as an occlusive device around the thigh with the aim of reducing blood flow distally. A survey in the United States found that 95% of surgeons used a tourniquet for TKR surgery. In the United Kingdom, over 90% of TKRs are performed with a tourniquet[3, 4]. Anecdotally surgeons believe using a tourniquet provides a bloodless field to improve the operative field of view[5]. Many surgeons also believe using a tourniquet improves the quality of the cementation of the knee implants[6], by reducing bone bleeding and allowing better inter digitation of the cement into the porous bone.

Previous systematic reviews have concluded that the use of tourniquets did not reduce intra or post-operative blood loss[7] and were associated with significant complications including venous thromboembolic events (VTE) [6], wound infection, bruising and nerve palsy[6, 8].

In TKR surgery, a tourniquet causes both arterial and venous stasis for the duration it is applied. It is therefore unsurprising that the use of a thigh tourniquet might increase the risk of post-operative VTE.[3] Potentially of much greater concern is that VTE may not be the only thromboembolic risk associated with using a tourniquet. Systemic emboli can occur following the deflation of a tourniquet.[9] Transoesophageal echocardiography has demonstrated shower-like echogenic materials circulating from the lower limbs to the right atrium, ventricle, and pulmonary artery after the release of a thigh tourniquet and also macroscopic emboli in the central circulation.[9] Trans-cranial Doppler ultrasound studies show a 60% prevalence of echogenic material in the Circle of Willis after a tourniquet is released and that micro embolism can occur even in the absence of a patent foramen ovale.[10] The most likely route for emboli in these circumstances is through the pulmonary capillaries or the opening of other pulmonary vessels.[10] The presence of cerebral emboli, which can cause cerebral damage, may explain the higher than expected prevalence of post-operative cognitive deficit following TKR. In the literature this varies from 41%–75% at 7 days to 18%–45% at 3 months post operatively.[11] These percentages are much higher than recorded in other major lower limb procedures with similar types of anaesthetic, but where a tourniquet is not used.[12]

Although studies have demonstrated that tourniquets do not substatially reduce blood loss and may increase complications, a review of these studies, by Alcelik et al, identified significant design flaws, including issues with randomisation, blinding and the absence of clearly defined outcome measures.[8] Furthermore, no controlled studies have addressed or quantified one of the most potentially serious risks associated with tourniquet, which are cerebral emboli, and any resultant cognitive impairment.

There may be problems with running a trial which involves recruiting patients who, once it is explained, may not be prepared to accept the potential risks of surgery with a tourniquet. Equally surgeons may be not willing to change surgical practice for a randomised trial.

We designed a feasibility study, which includes three separate but integrated projects:

A - Pilot randomised controlled trial (RCT)

B - Integrated qualitative research study

C - Retrospective multi-centre cohort study

The objective of the SAFE-TKR study is to establish whether a full randomised controlled trial evaluating tourniquets in knee replacement surgery is warranted and feasible.

A. Pilot randomised controlled trial

For peer review only

The primary objective of the pilot trial is to estimate recruitment, crossover and follow-up of patients for a full trial.

Secondary objectives:

i. Evaluate MRI for detecting post-operative ischaemic cerebral emboli, including an estimate of the size and direction of any effect.

ii. Evaluate tools for detecting postoperative cognitive impairment. These tools include mini mental state examination (MMSE), Montreal cognitive assessment (MoCA) and the Oxford cognitive scree (OCS), including an estimate of the size and direction of any effect.

iii. Evaluate other candidate primary/co-primary/secondary outcome measures for assessment within a larger trial including: thigh pain, symptomatic VTE, mortality, revision surgery, blood transfusion requirements, function and health related quality of life. To include obtaining estimates for the standard deviation of continuous outcome variables and differences in proportions for categorical outcome variables in order to facilitate potential sample size calculations for a full trial.

iv. Test and optimise patient information material and the patient pathway for a full trial.

Method

The protocol was produced in accordance with the Standard Protocol Items: Recommendations for Interventional Trials (SPIRIT) guidelines[13]. Ethical approval was obtained from Edgbaston Research Ethics Committee (15/WM/0455) on 27th January 2016. The trial will be reported in line with the CONSORT (Consolidated Standards of Reporting Trials) statement.[14]

A single-centre two arm pilot randomised controlled trial will be performed. All patients under the care of thirteen participating orthopaedic consultants at University Hospital Coventry and Warwickshire National Health Service (NHS) trust are potentially eligible for entry into the trial. The following eligibility criteria will be implemented for patient selection:

Inclusion criteria

i. Undergoing a primary unilateral/ total knee replacement

ii. Age ≥ 18

iii. Able to give written informed consent and to participate fully in the interventions and follow-up procedures

Exclusion criteria

i. Patients for whom MRI is contraindicated due to:

a. Non compliant heart pacemaker or defibrillator.

b. Non compliant metallic foreign body e.g. in one or both eyes and aneurysm clips in the brain.

c. Claustrophobia (e.g. difficulty in an elevator or telephone box).

ii. Patients not suitable for a thigh tourniquet (e.g. peripheral vascular disease).

iii. Previous participation in the SAFE-TKR study.

Patients already scheduled for total knee replacement surgery will be screened based on the eligibility criteria. A trained research associate will contact potentially eligible patients via telephone. Patients who are interested in taking part in the study will then be sent a patient information sheet and consent form and a date arranged to answer any further questions about the study and take consent. Verbal consent will initially be taken, followed by signed written consent via post or in person depending upon the patient's preference.

Randomisation

All patients who consent to the trial will be registered and then undergo a pre-op MRI of their brain. Patients will be allocated 1:1 via Warwick randomisation service (independent of the study team) to either TKR surgery with tourniquet or TKR surgery without tourniquet using minimisation to ensure balance between the treatment arms as regards patients with a previous history of VTE[15]. To ensure

For peer review only

allocation concealment, following enrolment patient details are entered on a web-based form and the treatment allocation generated.

Planned intervention
Patients will undergo routine elective primary unilateral TKR (cemented) using the standard technique of the anaesthetist and the operating surgeon. Both groups will have a thigh tourniquet applied to the relevant lower limb. Once the patient is fully anaesthetised one of the following interventions will be applied:
Group A (Tourniquet inflated)
The tourniquet will be inflated prior to the surgeon creating a wound and only deflated once the procedure is deemed completed by the surgeon (at a minimum this will be after all the TKR components have been finally inserted).
Group B (Tourniquet not inflated)
The tourniquet will not be inflated during the procedure.

In line with National Institute for Health and Care Excellence (NICE) guidance all patients will receive the following routine chemical and mechanical VTE prophylaxis:
i. Intermittent pneumatic calf compression until patient's mobility is no longer significantly reduced.
ii. Low molecular weight heparin (or unfractionated heparin for patients with severe renal impairment or established renal failure), started 6–12 hours after surgery and continued for 14 days post operatively.

Clinical outcomes and time points (see Table 1*)*
Primary clinical outcome
The primary clinical outcome will be evidence of new acute ischaemic brain lesions on MRI. The total number and volume of acute brain lesions detected on MRI per patient will be recorded. Pre- surgery MRIs will be obtained no more than 60 days before and post-surgery MRIs will be obtained up to 2 days after surgery. Diffuse weighted MRI is the most powerful tool for diagnosing acute ischaemic brain lesion caused by cerebral micro-embolism providing high level of sensitivity and specificity[16-18]. The MRIs will all follow a standardised protocol including fast spin echo-fluid attenuated inversion recovery sequences (FSE-FLAIR) and diffusion-weighted-spin echo-echo planar imaging (DWI SEEPI). The diffusion-weighted sequence will consist of an initial T2-weighted acquisition followed by a second acquisition with the application of diffusion-sensitizing gradients in the three orthogonal directions. Lesions will also be described according to the vascular territory (anterior, middle, posterior cerebral arteries, or vertebrobasilar arteries), side and type (cortical versus subcortical or deep grey matter). A planimetry of each lesion will be performed by using a grid overlay (using Image Processing and Analysis in JAVA software, National Institutes of Health), and calculating lesion volume by multiplying the number of involved grids by the slice thickness and slice gap. Attack rate (presence of new lesions/number of patients) will also be calculated. A new lesion will be defined as a focal hyper intense area detected by the fluid-attenuated inversion recovery sequence, corresponding to a restricted diffusion signal in the diffusion-weighted imaging sequence.[19] Scans will be read and evaluated by two experienced consultant radiologists blinded to the timing of the imaging, allocated intervention and the neurological status of the patient. Even asymptomatic erebral emboli, which may be detected using this approach, are associated with gradual memory impairment and cognitive decline.[20-22]

Secondary outcomes
1. Montreal Cognitive Assessment (MoCA) preoperatively and day 1, 2 and 1 week post operatively:
The MoCA is a brief cognitive screening tool with high sensitivity and specificity for detecting mild cognitive impairment[23].
2. Oxford Cognitive Screen (OCS) preoperatively and day 1, 2 and 1 week post operatively:

For peer review only - http://bmjopen.bmj.com/site/about/guidelines.xhtml

The OCS has higher sensitivity than the MOCA for measuring cognitive deficits associated with stroke (spatial disorders, apraxia) and it provides response speed as well as accuracy measures, so that indices such as processing speed can be derived.[24]

3. Mini-mental state examination (MMSE) scores preoperatively and day 1, 2 and 1 week post operatively:

The MMSE is the most commonly used tool for measuring cognitive impairment and has been used extensively to measure disturbances in post-operative cognition.[25-27]

4. Acute thigh pain preoperatively and on day 1, 2 and 1 week post operatively:

The 100mm VAS with 0 being no pain and 100mm being the worst pain is a validated patient reported outcome measure for pain following TKR surgery.[28]

5. Oxford Knee Score (OKS) preoperatively and at 1week, 6 and 12 months postoperatively:

This is a self-administered, validated knee replacement composite outcome measure of knee pain and function consisting of 12 items. The score ranges from 12 to 60, where 12 represents the best outcome and 60 represents the worst outcome.[29]

6. EQ-5D-5L scores preoperatively and at one week, six and 12 months postoperatively:

This is a validated measure of health-related quality of life, consisting of a five-dimension health status classification system and a separate visual analogue scale. EQ-5D-5L is primarily designed for self-completion by respondents and suited for use in postal surveys, in clinics and face-to-face interviews. It is cognitively simple, taking only a few minutes to complete.[30, 31]

7. Number of symptomatic VTE events up to 12 months postoperatively

Symptomatic VTE events will be defined based on NICE guidance: deep vein thrombosis = swollen or painful leg and a positive proximal leg vein ultrasound scan, pulmonary embolism = chest pain, shortness of breath or haemoptysis and positive Computed Topography Pulmonary Angiogram (CTPA) scan (or V/Q SPECT or planar scan if CTPA not available). Symptomatic VTEs will be captured throughout the post-operative period via hospital records. In addition, patient questionnaires at six and 12 months will capture further data.

8. Surgical complications up to 12 months postoperatively

Patient questionnaires and healthcare records will collect adverse events that are deemed to be as a direct result of surgery. Two blinded researchers will determine whether AEs should be classified as a surgical complication, where there is disagreement a third researcher will determine the final allocation.

9. Number of intra/post-operative blood transfusions until discharge

Data will be obtained from hospital records and recorded as number of units transfused.

10. Change in haemoglobin concentration [Hb g/L]

Routinely collected Haemoglobin concentrations [Hb g/L] measured from a full blood count taken on day 1 to 3 post operatively (the sample closest to day 1 will be favoured) will be subtracted from a preoperative [Hb g/L] measured within 3 months (the sample closest to the date of surgery will be favoured).

11. Revision rate of the TKR prosthesis at 12 months

Revision of the prosthesis for any reason will be established by patient questionnaires at 12 months and hospital records.

12. All-cause mortality rates at 12 months

Table 1 – Clinical outcomes and time points

Time Point	Data Collection
Baseline	Demographics, total volume of acute brain lesions, MMSE, MoCA, OCS, OKS, EQ-5D-5S, VAS, haemoglobin level.

Day 1	Total volume of acute brain lesions, MMSE, MoCA, OCS, VAS and haemoglobin level.
Day 2	Total volume of acute brain lesions (if not done on day 1), MMSE, MoCA, OCS, VAS and haemoglobin level (if not done on day 1).
Week 1	MMSE, MoCA, OCS, VA, OKS, EQ-5D, number of intra/post-operative blood transfusions until discharge and surgical complications
6 months	OKS, EQ-5D and surgical complications
12 months	OKS, EQ-5D-5L, number of symptomatic VTEs, surgical complications, revision rate and all cause mortality.

Data will be obtained from next of kin and healthcare records.
All baseline data will be summarised descriptively by intervention group. The flow of patients through the trial will be presented in a CONSORT diagram and patients withdrawn summarised by treatment group.

Assessment and blinding
It will not be possible to blind the clinicians administering the intervention. Patients and research associates collecting outcome measures will be blinded to treatment allocation. Patients with any outstanding postoperative cognitive tests (typically either day two or day seven) after discharge from hospital will have these administered by a trained research associate visiting them at home or an agreed location. Patients will receive a follow up telephone call at six weeks to record any VTE events since surgery. OKS, EQ-5D and complications questionnaire will be posted to the patient at 6 and 12 months. We will use techniques common in long-term cohort studies to ensure minimum loss to follow-up, such as collection of next of kin contact addresses and telephone numbers, mobile telephone numbers and email addresses. A maximum of three attempts will be made acquire outcome data at each time point.

Sample size calculation
As this is a pilot trial and not designed to measure effect a formal sample size calculation is not required (the statistical analysis will be largely descriptive). We propose seeking to recruit and obtain primary clinical outcome data for 50 patients for descriptive analysis.

Statistical analysis
Standard descriptive statistics (e.g. medians and ranges or means and variances, dependent on the distribution of the outcome) and graphical plots showing correlations will be presented for the primary and secondary outcome measures. Baseline data will be summarised to check comparability between treatment arms, and to highlight any characteristic differences between those individuals in the trial, those ineligible, and those eligible but withholding consent. Exploratory analyses using regression techniques will be used to assess change in total volume of acute brain lesions in the two treatment groups and investigate the relationship between cognitive test scores and total volume of acute brain lesions. Linear regression will also be used to estimate the proportion of the variation in total volume of acute brain lesions that may be explained by change in cognition score between baseline and first post-operative assessment. To further investigate the extent to which cognition score is a surrogate for total volume of acute brain lesions we will use logistic regression to predict treatment group from change in the total volume of acute brain lesions. The relationship between cognitive symptoms and the MRI data will also be evaluated using voxel-based lesion-symptom mapping, where MRI intensity changes are associated to cognitive deficits using either classification scores (deficit or not) or continuous measures of cognition.

For peer review only

The routine statistical analysis will mainly be carried out using STATA (Data Analysis and Statistical Software).

Data Management
After baseline demographic data is collected, a unique trial number will identify patients. All data collected will be entered into a secure trial database held at Warwick Clinical Trials Unit (WCTU). Identifiable patient information will be held in a locked filing cabinet and coded with a patient trial number. The WCTU quality assurance manager will audit trial records in accordance with Standard Operating Procedures. Outcomes will not be analysed until all primary outcome data is collected.

Trial oversight
The Trial Management Group, consisting of the staff involved in the day-to-day running of the study, will meet monthly. Significant issues arising from management meetings will be referred to the Trial Steering Committee as appropriate. The trial will be guided by a Trial Steering Committee (TSC), a group of respected and experienced personnel and trialists as well as lay representatives. The TSC will have an independent Chairperson. At least two formal TSC meeting will be held, one before the trial starts and one before recruitment to the trial completes. As this is a small feasibility study there will not be a data monitoring committee. Adverse events and SAE's will be monitored by the investigators. Adverse events will be assessed for causality within 24 hours of notification and patients followed up as per protocol. The trial may be stopped prematurely if mandated by ethics committee, a major unexpected safety concern arises or funding ceases.

B - Integrated qualitative research study

Method
Patients
In depth semi-structured interviews amongst with randomised patients and potential patients who decline to take part in the pilot trial will help understand people's views regarding participation in the pilot trial. A purposive sample of around 30 people (evenly split between recruited and not recruited) will be interviewed, based on patient demographics (including age, gender and socioeconomic status).[32, 33] Patients will be recruited by a trained research associate and interviews will then be undertaken at a time convenient to the patient.

Surgeons
A survey will be undertaken amongst surgeons who routinely do TKR surgery. The survey will help gauge the extent to which this community is in clinical equipoise and would be willing to engage with, and support a larger trial. We will use a web-based survey of members of the British Association for Surgery of the Knee (BASK). BASK has a UK membership of over 100 practising knee surgeons of whom the majority routinely undertake TKR surgery. A sample of at least 20 BASK surgeons (both those clearly in equipoise and those that are not) will be invited to take part in more in depth qualitative interviews. We will use the general theory of implementation to investigate and understand the core constructs of sense making processes of patients and surgeons regarding the feasibility of a full trial and to help understand any barriers and enablers for a larger trial.

Digital audio recordings of interviews will be transcribed verbatim, checked and anonymised. Data will be managed and shared using N-Vivo analysis software. The analysis will be informed by a constant comparative approach, where early analysis informs subsequent data collection, and data analysis takes place alongside data collection.[32] This means insights from patients and surgeons can be explored further in the on-going interviews. Data is coded from the start of data collection, and new data is compared with existing data. Codes are compared, categories are constructed and explored to ensure they are robust and then they are linked with relevant theoretical literature. A technique of

For peer review only - http://bmjopen.bmj.com/site/about/guidelines.xhtml

triangulation protocol will be used to facilitate the integration of the findings of the qualitative research with the quantitative data thereby helping to determine the overall feasibility of a full trial.[34] The data from patient interviews will also be used to help design optimal patient information materials for a larger trial.

C - Multi-centre cohort study

The National Joint Registry (NJR) is a population-based registry of joint replacements in the UK and covers both the NHS and private sectors. From April 2003 to December 2003 the NJR collected data on the use of tourniquets for TKR surgery in England and Wales. At this stage there were 406 hospitals listed within the NJR system (NHS hospitals, independent sector hospitals, and Treatment Centres - both NHS funded and privately funded) and of these, 384 returned data (i.e. 94%).The NJR dataset also contains many other key variables which are known to affect mortality, implant survivorship (revision rate) and the risk of VTE (1) use and type of VTE prophylaxis (such as low molecular weight heparin, aspirin, intermittent calf pump) (2) type of implant used (3) use of cement (4) basic patient demographics including age, body mass index (BMI) and American Society of Anaesthesiologists (ASA) grade.

Method

We will access NJR data for the period in which tourniquet use was collected as part of the minimum dataset standard for knee replacement surgery – April 2003 to December 2003. We will analyse only data from primary cemented TKRs. Tourniquet use along with other routinely collected NJR baseline variables: age at time of surgery, sex, American Society of Anaesthesiologists (ASA) classification will be analysed to measure for independent effects on all cause revision. A tourniquet will be classed as used if even for a selective period of the procedure such as cementation. Sociodemographics, co-morbidities, VTEs, cerebrovascular accident (CVAs or stroke) and length of hospital stay will be obtained by using patient identifiers (NHS Number, gender, date of birth, postcode, procedure code and local patient identifier code) from the NJR to link the data to the Hospital Episode Statistics (HES) database. The HES data will provide details of VTE, socio-demographics, comorbidities and hospital length of stay for the TKR. The study will specifically estimate differences between groups (tourniquet or no tourniquet) for the following:

i. Baseline characteristics (age, BMI, ASA, co-morbidities, type of VTE prophylaxis, type of implant, socio-demographics)
ii. All cause mortality up to 12 months
iii. Length of inpatient hospital stay
iv. Risk of surgical complications up to 30 days
v. Risk of VTE up to 12 months
vi. Risk of CVA up to 12 months
vii. Risk of revision at 1, 5 and 10 years

Statistical Analysis

The Kaplan-Meier method will be used to calculate mortality and revision rates in those who underwent TKR with and without a tourniquet. Multivariate logistic regression will be used to assess the effect of age, body mass index, socio-economic status, tourniquet use, type of VTE prophylaxis, American Society of Anaesthesiologists (ASA) grade, co-morbidities and type of TKR implant (cemented or uncemented) on risk of VTE, CVA and risk of revision. The results of these analyses will inform the planning of a subsequent definitive full trial, if such a trial is feasible.

Conclusions - safety and feasibility evaluation and pathway to a full trial

A workshop will be held at the end of the feasibility study, involving key stakeholders (approximately 30 participants), patients, surgeons, researchers, allied healthcare professionals and healthcare policy makers. The workshop will have an independent chairperson. Using consensus conference

methodology, delegates will be presented with results from the pilot RCT, retrospective multi-centre cohort study, existing published data and where applicable pooled data from all three sources. The purpose will be to agree a consensus statement on the appropriateness and feasibility of proceeding to a full trial and the most appropriate primary outcome measure for a full trial.

A full definitive trial will be deemed feasible if:

i. The pilot RCT and integrated qualitative study suggest patients can be recruited and surgeons are prepared to perform surgery with or without a tourniquet**.**

AND

ii. The pilot trial identifies either a measure of cognitive brain function that accurately reflects symptomatic brain emboli or an alternative primary/co-primary outcome measure is identified such as pain, symptomatic VTE, revision rates, OKS or EQ5D-5L scores that can be accurately and robustly collected in a full trial.

AND

iii. Key stakeholders (patients, surgeons, public representatives and researchers) agree at an end of study consensus conference that in light of the feasibility study and existing published research there remains insufficient data to make recommendations about the safety and clinical effectiveness of tourniquets and that a full trial is feasible and there is an appropriate primary / co-primary outcome measure for assessment.

Acknowledgments, Competing Interests, Funding and other required statements

Acknowledgements

We would also like to acknowledge the support of Warwick Clinical Trials unit and University Hospital Coventry and Warwickshire.
The operating surgeons involved in this study include: A Ali, M Blakemore, K El-Bayouk, P Foguet, A Kotecha, R King, J McArthur, A Metcalfe, M Margetts, S Patil, B Riemer, K Sarantos, F Shah, T Spalding, P Thompson, R Westerman.

Competing Interests:

All authors have no competing interests to declare.

Ethics and Dissemination

The study trial obtained approval from the National Research Ethics Committee (NRES) West Midlands – Edgbaston (15/WM/0455) on the 27th January 2016.
We aim to publish the results in at least one high impact peer reviewed journal. The results of the trial will also be disseminated via patient information material produced in collobroation with our Puplic Patient Involvement group. All key study findings will be presented at national and international conferences e.g. British Orthopaedic Association (BOA), British Association of Specialist Knee Surgeons (BASK) and America Academy of Orthopedic Surgeons (AAOS).

Funding and sponsorship

The study protocol represents research funded by a National Institute for Health Research (NIHR) Post-Doctoral Fellowship Award (PDF-2015-08-108). The study is jointly sponsored by the University of Warwick and University Hospitals Coventry & Warwickshire NHS Trust. The trial sponsors provide ultimate approval of all new versions of the protocol before they become live. Both the funders and sponsors are required to provide final approval before publication of any study material.

Author Contributions

For peer review only

- PW: Chief investigator, study conception, study design, data collection, drafted and reviewed final manuscript
- IA: Data collection, drafted and reviewed final manuscript
- AM: Principal investigator, drafted and reviewed final manuscript
- AP: Study design, drafted and reviewed final manuscript
- KS: Study design, drafted and reviewed final manuscript
- CH: Study design, data analysis, drafted and reviewed final manuscript
- HP: Data analysis, drafted and reviewed final manuscript
- JW: Data analysis, drafted and reviewed final manuscript
- BR: Trial manager, data collection, drafted and reviewed final manuscript
- JB: Trial manager, data collection, drafted and reviewed final manuscript
- MU: Study design, drafted and reviewed final manuscript
- SAFE-TKR Study Group: Study design and reviewed final manuscript.

Collaborators

SAFE-TKR Study Group: B Rahman, J Brown, C Goulden, K Seers, A Metcalfe, J Smith, J Dixon, J Warwick, H Parsons, N Demeyere, C Hutchinson, I Ahmed, A Price, M Underwood, P Wall.

References

1. Murphy L, Schwartz TA, Helmick CG, Renner JB, Tudor G, Koch G, et al. Lifetime risk of symptomatic knee osteoarthritis. Arthritis and rheumatism. 2008 Sep 15;59(9):1207-13.
2. Callahan CM, Drake BG, Heck DA, Dittus RS. Patient outcomes following tricompartmental total knee replacement. A meta-analysis. Jama. 1994 May 4;271(17):1349-57. Epub 1994/05/04.
3. Zhang W, Li N, Chen S, Tan Y, Al-Aidaros M, Chen L. The effects of a tourniquet used in total knee arthroplasty: a meta-analysis. Journal of orthopaedic surgery and research. 2014;9(1):13.
4. National Joint Registry for England and Wales. National Joint Registry for England and Wales 1st Annual Report 2004.
5. Parvizi J, Diaz-Ledezma C. Total knee replacement with the use of a tourniquet: more pros than cons. The bone & joint journal. 2013 Nov;95-B(11 Suppl A):133-4.
6. Tai TW, Lin CJ, Jou IM, Chang CW, Lai KA, Yang CY. Tourniquet use in total knee arthroplasty: a meta-analysis. Knee surgery, sports traumatology, arthroscopy : official journal of the ESSKA. 2011 Jul;19(7):1121-30.
7. Smith TO, Hing CB. Is a tourniquet beneficial in total knee replacement surgery? A meta-analysis and systematic review. The Knee. 2010 Mar;17(2):141-7.
8. Alcelik I, Pollock RD, Sukeik M, Bettany-Saltikov J, Armstrong PM, Fismer P. A comparison of outcomes with and without a tourniquet in total knee arthroplasty: a systematic review and meta-analysis of randomized controlled trials. The Journal of arthroplasty. 2012 Mar;27(3):331-40.
9. Berman AT, Parmet JL, Harding SP, Israelite CL, Chandrasekaran K, Horrow JC, et al. Emboli observed with use of transesophageal echocardiography immediately after tourniquet release during total knee arthroplasty with cement. The Journal of bone and joint surgery American volume. 1998 Mar;80(3):389-96.
10. Sulek CA, Davies LK, Enneking FK, Gearen PA, Lobato EB. Cerebral microembolism diagnosed by transcranial Doppler during total knee arthroplasty: correlation with transesophageal echocardiography. Anesthesiology. 1999 Sep;91(3):672-6. Epub 1999/09/15.
11. Deo H, West G, Butcher C, Lewis P. The prevalence of cognitive dysfunction after conventional and computer-assisted total knee replacement. The Knee. 2011 Mar;18(2):117-20. Epub 2010/07/10.
12. Koch S, Forteza A, Lavernia C, Romano JG, Campo-Bustillo I, Campo N, et al. Cerebral fat microembolism and cognitive decline after hip and knee replacement. Stroke; a journal of cerebral circulation. 2007 Mar;38(3):1079-81.

For peer review only

13. Chan AW, Tetzlaff JM, Altman DG, Laupacis A, Gotzsche PC, Krleza-Jeric K, et al. SPIRIT 2013 statement: defining standard protocol items for clinical trials. Annals of internal medicine. 2013 Feb 05;158(3):200-7. Epub 2013/01/09.
14. Schulz KF, Altman DG, Moher D, Group C. CONSORT 2010 statement: updated guidelines for reporting parallel group randomised trials. International journal of surgery. 2011;9(8):672-7.
15. Altman DG, Bland JM. Treatment allocation by minimisation. Bmj. 2005 Apr 9;330(7495):843.
16. Fairbairn TA, Mather AN, Bijsterveld P, Worthy G, Currie S, Goddard AJ, et al. Diffusion-weighted MRI determined cerebral embolic infarction following transcatheter aortic valve implantation: assessment of predictive risk factors and the relationship to subsequent health status. Heart. 2012 Jan;98(1):18-23.
17. Ghanem A, Muller A, Nahle CP, Kocurek J, Werner N, Hammerstingl C, et al. Risk and fate of cerebral embolism after transfemoral aortic valve implantation: a prospective pilot study with diffusion-weighted magnetic resonance imaging. Journal of the American College of Cardiology. 2010 Apr 6;55(14):1427-32.
18. Gress DR. The problem with asymptomatic cerebral embolic complications in vascular procedures: what if they are not asymptomatic? Journal of the American College of Cardiology. 2012 Oct 23;60(17):1614-6.
19. Bijuklic K, Wandler A, Tubler T, Schofer J. Impact of asymptomatic cerebral lesions in diffusion-weighted magnetic resonance imaging after carotid artery stenting. JACC Cardiovascular interventions. 2013 Apr;6(4):394-8.
20. Blum S, Luchsinger JA, Manly JJ, Schupf N, Stern Y, Brown TR, et al. Memory after silent stroke: hippocampus and infarcts both matter. Neurology. 2012 Jan 3;78(1):38-46.
21. Arvanitakis Z, Leurgans SE, Barnes LL, Bennett DA, Schneider JA. Microinfarct pathology, dementia, and cognitive systems. Stroke; a journal of cerebral circulation. 2011 Mar;42(3):722-7.
22. Karoutas G, Milonas I, Artemis N, Karacostas D, Pitas J, Logothetis J. The pharmacological effects of buflomedil in patients with multi-cerebral infarcts dementia: an open, preliminary assessment. Current medical research and opinion. 1987;10(6):380-9.
23. Stolwyk RJ, O'Neill MH, McKay AJ, Wong DK. Are cognitive screening tools sensitive and specific enough for use after stroke? A systematic literature review. Stroke; a journal of cerebral circulation. 2014 Oct;45(10):3129-34.
24. Demeyere N, Slavkova E, Riddoch MJ, Humphreys GW. The Oxford Cognitive Screen (OCS): Validation of a stroke-specific short cognitive screening tool. Psychological Assessment. 2015 (in press).
25. Petersen RC, Smith GE, Waring SC, Ivnik RJ, Tangalos EG, Kokmen E. Mild cognitive impairment: clinical characterization and outcome. Archives of neurology. 1999 Mar;56(3):303-8.
26. Saczynski JS, Marcantonio ER, Quach L, Fong TG, Gross A, Inouye SK, et al. Cognitive trajectories after postoperative delirium. The New England journal of medicine. 2012 Jul 5;367(1):30-9.
27. Folstein MF, Folstein SE, McHugh PR. "Mini-mental state". A practical method for grading the cognitive state of patients for the clinician. Journal of psychiatric research. 1975 Nov;12(3):189-98.
28. Marques EM, Jones HE, Elvers KT, Pyke M, Blom AW, Beswick AD. Local anaesthetic infiltration for peri-operative pain control in total hip and knee replacement: systematic review and meta-analyses of short- and long-term effectiveness. BMC musculoskeletal disorders. 2014;15:220.
29. Dawson J, Fitzpatrick R, Murray D, Carr A. Questionnaire on the perceptions of patients about total knee replacement. The Journal of bone and joint surgery British volume. 1998 Jan;80(1):63-9.
30. EuroQol G. EuroQol--a new facility for the measurement of health-related quality of life. Health policy. 1990 Dec;16(3):199-208.
31. Walters SJ, Brazier JE. Comparison of the minimally important difference for two health state utility measures: EQ-5D and SF-6D. Quality of life research : an international journal of quality of life aspects of treatment, care and rehabilitation. 2005 Aug;14(6):1523-32.

For peer review only

32. Charmaz KC. Constructing grounded theory: A practical guide through qualitative analysis2006.
33. Marshall B, Cardon P, Poddar A, Fontenot R. DOES SAMPLE SIZE MATTER IN QUALITATIVE RESEARCH?: A REVIEW OF QUALITATIVE INTERVIEWS IN IS RESEARCH. Journal of Computer Information Systems. 2013.
34. O'Cathain A, Murphy E, Nicholl J. Three techniques for integrating data in mixed methods studies. Bmj. 2010;341:c4587.

For peer review only

BMJ Open

Safety and feasibility evaluation of tourniquets for total knee replacement (SAFE- TKR): study protocol

Journal:	*BMJ Open*
Manuscript ID	bmjopen-2018-022067.R1
Article Type:	Protocol
Date Submitted by the Author:	22-Feb-2018
Complete List of Authors:	Wall, Peter ; University of Warwick, Clinical Trials Unit<br>Ahmed, Imran; University Hospital Coventry<br>Metcalfe, Andrew; University of Warwick, Clinical Trials Unit, Warwick Medical School<br>Price, Andrew; University of Oxford, Nuffield Department of Orthopaedics, Rheumatology and Musculoskeletal Sciences<br>Seers, Kate; Royal College of Nursing Research Institute, Warwick Medical School, University of Warwick, RCN RI<br>Hutchinson, Charles; University of Warwick, Warwick Medical School<br>Parsons, Helen; University of Warwick, Division of Health Sciences<br>Warwick, Jane; University of Warwick Warwick Medical School, Warwick Clinical Trials Unit<br>Rahman, Bushra; University of Warwick, Clinical Trials Unit<br>Brown, Jaclyn; University of Warwick, Clinical Trials Unit<br>Underwood, Martin; Warwick University, Warwick Medical School<br>SAFE-TKR study group , On behalf of; University of Warwick, Clinical Trials Unit
**Primary Subject Heading**:	Surgery
Secondary Subject Heading:	Surgery
Keywords:	Knee < ORTHOPAEDIC & TRAUMA SURGERY, Stroke < NEUROLOGY, Adult surgery < SURGERY

SCHOLARONE™
Manuscripts

Safety and feasibility evaluation of tourniquets for total knee replacement (SAFE- TKR): study protocol

Authors

Peter DH Wall, Imran Ahmed, Andrew Metcalfe, Andrew Price, Kate Seers, Charles E Hutchinson, Helen Parsons, Jane Warwick, Bushra Rahman, Jaclyn Brown, Martin Underwood and the SAFE-TKR Study Group.

Corresponding author

Mr Peter David Henry Wall

Clinical Lecturer in Trauma and Orthopaedic Surgery

Warwick Clinical Trials Unit

Warwick Medical School

University of Warwick

Coventry

CV4 7AL

p.d.h.wall@warwick.ac.uk

Peter Wall: Warwick Clinical Trials Unit, University of Warwick, Coventry, UK, CV4 7AL

Imran Ahmed: University Hospital Coventry Hospital, Clifford Bridge Road, Coventry, UK, CV2 2DX.

Andrew Metcalfe: Warwick Clinical Trials Unit, University of Warwick, Coventry, UK, CV4 7AL

Andrew Price: Nuffield Department of Orthopaedics, Rheumatology and Musculoskeletal sciences, Oxford, UK

Kate Seers: Royal College of Nursing Research Institute, University of Warwick, Coventry, UK, CV4 7AL

Charles Hutchinson: Warwick Medical School, University of Warwick Coventry, UK, CV2 2DX.

Helen Parsons: Warwick Clinical Trials Unit, University of Warwick, Coventry, UK, CV4 7AL

Jane Warwick: Warwick Clinical Trials Unit, University of Warwick, Coventry, UK, CV4 7AL

Bushra Rahman: Warwick Clinical Trials Unit, University of Warwick, Coventry, UK, CV4 7AL

Jaclyn Brown: Warwick Clinical Trials Unit, University of Warwick, Coventry, UK, CV4 7AL

For peer review only

For peer review only - http://bmjopen.bmj.com/site/about/guidelines.xhtml

Martin Underwood: Warwick Clinical Trials Unit, University of Warwick, Coventry, UK, CV4 7AL

SAFE-TKR Study group: Warwick Clinical Trials Unit, University of Warwick, Coventry, UK, CV4 7AL

Word count: 4201

For peer review only

Abstract for protocol

Introduction

This study is designed to determine whether a full randomised controlled trial (RCT) examining the clinical effectiveness and safety of total knee replacement (TKR) surgery with or without a tourniquet is warranted and feasible.

For peer review only - http://bmjopen.bmj.com/site/about/guidelines.xhtml

Method and analysis

Single centre, patient and assessor blinded RCT. A computer generated randomisation service will allocate 50 participants into one of two trial treatments, surgery with or without a tourniquet. The primary objective is to estimate recruitment, crossovers and followup of patients. All patients will have a Magnetic Resonance Imaging (MRI) scan of their brain preoperatively and day 1 or 2 postoperatively to identify ischaemic cerebral emboli (primary clinical outcome). Oxford Cognitive Screen, Montreal Cognitive Assessment, Mini-Mental State Examination will be evaluated as outcome tools for measuring cognitive impairment at day 1, 2 and 7 postoperatively. Thigh pain, blood transfusion requirements, venous thromboembolism, revision surgery, surgical complications, mortality and Oxford knee and EQ-5D-5L Scores will be collected over 12 months.

Integrated qualitative research study: 30 trial patients and 20 knee surgeons will take part in semi-structured interviews. Interviews will capture views regarding the pilot trial and explore barriers and potential solutions to a full trial.

Multi-centre cohort study: UK National Joint Registry (NJR) data will be linked to Hospital Episode Statistics (HES) to estimate the relationship between tourniquet use and VTE, length of hospital stay, risk of revision surgery and death.

The study will conclude with a multidisciplinary workshop to reach a consensus on whether a full trial is warranted and feasible.

Ethics and dissemination

National Research Ethics Committee (West Midlands-Edgbaston) approved this study on 27/01/2016 (15/WM/0455). The study is sponsored by University of Warwick and University Hospitals Coventry and Warwickshire. The results will be disseminated via high impact peer-reviewed publication.

Funding: National Institute for Health Research Post-Doctoral Fellowship Award (NIHR-PDF-2015-08-108).

Trial Registration: ISRCTN20873088

Article Summary

Strengths and limitations of the study

Strengths

- Comprehensive feasibility research
- Clearly defined outcome measures
- Patient blinding

Limitations

For peer review only - http://bmjopen.bmj.com/site/about/guidelines.xhtml

- Single centre design with small sample size
- Absence of blinding among clinicians delivering the intervention

For peer review only

Introduction

Arthritis of the knee, can cause pain and restrict activities of daily living. Total knee replacement (TKR) surgery is a surgical procedure aimed at resolving the symptoms of end stage knee arthritis[1]. TKR surgery is typically undertaken with the aid of a tourniquet. A tourniquet acts as an occlusive device around the thigh with the aim of reducing blood flow distally. In the United Kingdom, over 90% of TKRs are performed with a tourniquet[2,3]. Anecdotally surgeons believe using a tourniquet provides a bloodless field to improve the operative field of view[4]. Many surgeons also believe using a tourniquet improves the quality of the cementation of the knee implants[5], by reducing bone bleeding and allowing better inter digitation of the cement into the porous bone.

Previous systematic reviews have concluded that the use of tourniquets did not reduce intra or post-operative blood loss[6] and were associated with significant complications including venous thromboembolic events (VTE) [5], wound infection, bruising and nerve palsy.[5,7]

In TKR surgery, a tourniquet causes both arterial and venous stasis for the duration it is applied. It is therefore unsurprising that the use of a thigh tourniquet might increase the risk of post-operative VTE.[2] However, VTE may not be the only thromboembolic risk associated with using a tourniquet. Research has demonstrated that systemic emboli can occur when the tourniquet is deflated with upto a 60% prevalence of echogenic material in the Circle of Willis .[8,9] Emboli may reach the systemic circulation through the pulmonary capillaries or the opening of other pulmonary vessels.[9] The prevalence of post operative cognitive impairment after TKR is high with reports In the literature varying from 41%–75% at 7 days to 18%–45% at 3 months, and this may in part be explained by cerebral emboli if they are occurring following the release of a tourniquet.[10]

Although studies have demonstrated that tourniquets do not substatially reduce blood loss and may increase complications, a review of these studies, by Alcelik et al, identified significant design flaws, including issues with randomisation, blinding and the absence of clearly defined outcome measures.[7]

For peer review only - http://bmjopen.bmj.com/site/about/guidelines.xhtml

Furthermore, no controlled studies have addressed or quantified one of the most potentially serious risks associated with tourniquet, which are cerebral emboli, and any resultant cognitive impairment.

There may be problems with running a trial which involves recruiting patients who, once it is explained, may not be prepared to accept the potential risks of surgery with a tourniquet. Equally surgeons may be not willing to change surgical practice for a randomised trial.

We designed a feasibility study, which includes three separate but integrated projects:

A - Pilot randomised controlled trial (RCT)

B - Integrated qualitative research study

C - Retrospective multi-centre cohort study

The objective of the SAFE-TKR study is to establish whether a full randomised controlled trial evaluating tourniquets in knee replacement surgery is warranted and feasible.

A. Pilot randomised controlled trial

The primary objective of the pilot trial is to estimate recruitment, crossover and follow-up of patients for a full trial.

Secondary objectives:

i. Evaluate MRI for detecting post-operative ischaemic cerebral emboli, including an estimate of the size and direction of any effect.
ii. Evaluate tools for detecting postoperative cognitive impairment. These tools include mini mental state examination (MMSE), Montreal cognitive assessment (MoCA) and the Oxford cognitive scree (OCS), including an estimate of the size and direction of any effect.
iii. Evaluate other candidate primary/co-primary/secondary outcome measures for assessment within a larger trial including: thigh pain, symptomatic VTE, mortality, revision surgery, blood transfusion requirements, function and health related quality of life. To include obtaining estimates for the standard deviation of continuous outcome variables and differences in proportions for categorical outcome variables in order to facilitate potential sample size calculations for a full trial.
iv. Test and optimise patient information material and the patient pathway for a full trial.

Method

The protocol was produced in accordance with the Standard Protocol Items: Recommendations for Interventional Trials (SPIRIT) guidelines[11]. Ethical approval was obtained from Edgbaston Research Ethics

For peer review only - http://bmjopen.bmj.com/site/about/guidelines.xhtml

Committee (15/WM/0455) on 27th January 2016. The trial will be reported in line with the CONSORT (Consolidated Standards of Reporting Trials) statement.[12]

A single-centre two arm pilot randomised controlled trial will be performed. All patients under the care of thirteen participating orthopaedic consultants at University Hospital Coventry and Warwickshire National Health Service (NHS) trust are potentially eligible for entry into the trial. The following eligibility criteria will be implemented for patient selection:

Inclusion criteria

i. Undergoing a primary unilateral/ total knee replacement

ii. Age ≥ 18

iii. Able to give written informed consent and to participate fully in the interventions and follow-up procedures

Exclusion criteria

i. Patients for whom MRI is contraindicated due to:
   a. Non compliant heart pacemaker or defibrillator.
   b. Non compliant metallic foreign body e.g. in one or both eyes and aneurysm clips in the brain.
   c. Claustrophobia (e.g. difficulty in an elevator or telephone box).
ii. Patients not suitable for a thigh tourniquet (e.g. peripheral vascular disease).
iii. Previous participation in the SAFE-TKR study.

Patients already scheduled for total knee replacement surgery will be screened based on the eligibility criteria. A trained research associate will contact potentially eligible patients via telephone. Patients who are interested in taking part in the study will then be sent a patient information sheet and consent form and a date arranged to answer any further questions about the study and take consent. Verbal consent will initially be taken, followed by signed written consent via post or in person depending upon the patient's preference, see appendix 1 and 2.

Randomisation

All patients who consent to the trial will be registered and then undergo a pre-op MRI of their brain. Patients will be allocated 1:1 via Warwick randomisation service (independent of the study team) to either TKR surgery with tourniquet or TKR surgery without tourniquet using minimisation to ensure balance between the treatment arms as regards patients with a previous history of VTE[13]. To ensure allocation concealment, following enrolment patient details are entered on a web-based form and the treatment allocation generated.

For peer review only - http://bmjopen.bmj.com/site/about/guidelines.xhtml

Planned intervention

Patients will undergo routine elective primary unilateral TKR (cemented) using the standard technique of the anaesthetist and the operating surgeon. Both groups will have a thigh tourniquet applied to the relevant lower limb. Once the patient is fully anaesthetised one of the following interventions will be applied:

Group A (Tourniquet inflated)

The tourniquet will be inflated prior to the surgeon creating a wound and only deflated once the procedure is deemed completed by the surgeon (at a minimum this will be after all the TKR components have been finally inserted).

Group B (Tourniquet not inflated)

The tourniquet will not be inflated during the procedure.

In line with National Institute for Health and Care Excellence (NICE) guidance all patients will receive the following routine chemical and mechanical VTE prophylaxis:

i. Intermittent pneumatic calf compression until patient's mobility is no longer significantly reduced.
ii. Low molecular weight heparin (or unfractionated heparin for patients with severe renal impairment or established renal failure), started 6–12 hours after surgery and continued for 14 days post operatively.

Clinical outcomes and time points (see Table 1*)*

Primary clinical outcome

The primary clinical outcome will be evidence of new acute ischaemic brain lesions on MRI. The total number and volume of acute brain lesions detected on MRI per patient will be recorded. Pre- surgery MRIs will be obtained no more than 60 days before and post-surgery MRIs will be obtained up to 2 days after surgery. Diffuse weighted MRI is the most powerful tool for diagnosing acute ischaemic brain lesion caused by cerebral micro-embolism providing high level of sensitivity and specificity[14-16]. The MRIs will all follow a standardised protocol including fast spin echo-fluid attenuated inversion recovery sequences (FSE-FLAIR) and diffusion-weighted-spin echo-echo planar imaging (DWI SEEPI). The diffusion-weighted sequence will consist of an initial T2-weighted acquisition followed by a second acquisition with the application of diffusion-sensitizing gradients in the three orthogonal directions. Lesions will also be described according to the vascular territory (anterior, middle, posterior cerebral arteries, or vertebrobasilar arteries), side and type (cortical versus subcortical or deep grey matter). A planimetry of each lesion will be performed by using a grid overlay (using Image Processing and Analysis in JAVA software, National Institutes of Health), and calculating lesion volume by multiplying the number of involved grids by the slice thickness and slice gap. Attack rate (presence of new lesions/number of patients) will also be calculated. A new lesion will be defined as a focal hyper intense

For peer review only - http://bmjopen.bmj.com/site/about/guidelines.xhtml

area detected by the fluid-attenuated inversion recovery sequence, corresponding to a restricted diffusion signal in the diffusion-weighted imaging sequence.[17] Scans will be read and evaluated by two experienced consultant radiologists blinded to the timing of the imaging, allocated intervention and the neurological status of the patient. Even asymptomatic erebral emboli, which may be detected using this approach, are associated with gradual memory impairment and cognitive decline.[18-20]

Secondary outcomes

1. Montreal Cognitive Assessment (MoCA) preoperatively and day 1, 2 and 1 week post operatively:
The MoCA is a brief cognitive screening tool with high sensitivity and specificity for detecting mild cognitive impairment[21].

2. Oxford Cognitive Screen (OCS) preoperatively and day 1, 2 and 1 week post operatively:
The OCS has higher sensitivity than the MOCA for measuring cognitive deficits associated with stroke (spatial disorders, apraxia) and it provides response speed as well as accuracy measures, so that indices such as processing speed can be derived.[22]

3. Mini-mental state examination (MMSE) scores preoperatively and day 1, 2 and 1 week post operatively:
The MMSE is the most commonly used tool for measuring cognitive impairment and has been used extensively to measure disturbances in post-operative cognition.[23-25]

4. Acute thigh pain preoperatively and on day 1, 2 and 1 week post operatively:
The 100mm VAS with 0 being no pain and 100mm being the worst pain is a validated patient reported outcome measure for pain following TKR surgery.[26]

5. Oxford Knee Score (OKS) preoperatively and at 1week, 6 and 12 months postoperatively:
This is a self-administered, validated knee replacement composite outcome measure of knee pain and function consisting of 12 items. The score ranges from 12 to 60, where 12 represents the best outcome and 60 represents the worst outcome.[27]

6. EQ-5D-5L scores preoperatively and at one week, six and 12 months postoperatively:
This is a validated measure of health-related quality of life, consisting of a five-dimension health status classification system and a separate visual analogue scale. EQ-5D-5L is primarily designed for self-completion by respondents and suited for use in postal surveys, in clinics and face-to-face interviews. It is cognitively simple, taking only a few minutes to complete.[28,29]

7. Number of symptomatic VTE events up to 12 months postoperatively
Symptomatic VTE events will be defined based on NICE guidance: deep vein thrombosis = swollen or painful leg and a positive proximal leg vein ultrasound scan, pulmonary embolism = chest pain, shortness of breath or haemoptysis and positive Computed Topography Pulmonary Angiogram (CTPA) scan (or V/Q SPECT or planar scan if CTPA not available). Symptomatic VTEs will be captured throughout the post-operative period via hospital records. In addition, patient questionnaires at six and 12 months will capture further data.

8. Surgical complications up to 12 months postoperatively

For peer review only - http://bmjopen.bmj.com/site/about/guidelines.xhtml

For peer review only

Patient questionnaires and healthcare records will collect adverse events that are deemed to be as a direct result of surgery. Two blinded researchers will determine whether AEs should be classified as a surgical complication, where there is disagreement a third researcher will determine the final allocation.

9. Number of intra/post-operative blood transfusions until discharge
Data will be obtained from hospital records and recorded as number of units transfused.

10. Change in haemoglobin concentration [Hb g/L]
Routinely collected Haemoglobin concentrations [Hb g/L] measured from a full blood count taken on day 1 to 3 post operatively (the sample closest to day 1 will be favoured) will be subtracted from a preoperative [Hb g/L] measured within 3 months (the sample closest to the date of surgery will be favoured).

11. Revision rate of the TKR prosthesis at 12 months
Revision of the prosthesis for any reason will be established by patient questionnaires at 12 months and hospital records.

12. All-cause mortality rates at 12 months

Table 1 – Clinical outcomes and time points

Time Point	Data Collection
Baseline	Demographics, total volume of acute brain lesions, MMSE, MoCA, OCS, OKS, EQ-5D-5S, VAS, haemoglobin level.
Day 1	Total volume of acute brain lesions, MMSE, MoCA, OCS, VAS and haemoglobin level.
Day 2	Total volume of acute brain lesions (if not done on day 1), MMSE, MoCA, OCS, VAS and haemoglobin level (if not done on day 1).
Week 1	MMSE, MoCA, OCS, VA, OKS, EQ-5D, number of intra/post-operative blood transfusions until discharge and surgical complications
6 months	OKS, EQ-5D and surgical complications
12 months	OKS, EQ-5D-5L, number of symptomatic VTEs, surgical complications, revision rate and all cause mortality.

Data will be obtained from next of kin and healthcare records.

All baseline data will be summarised descriptively by intervention group. The flow of patients through the trial will be presented in a CONSORT diagram and patients withdrawn summarised by treatment group.

Assessment and blinding

For peer review only - http://bmjopen.bmj.com/site/about/guidelines.xhtml

It will not be possible to blind the clinicians administering the intervention. Patients and research associates collecting outcome measures will be blinded to treatment allocation. Patients with any outstanding postoperative cognitive tests (typically either day two or day seven) after discharge from hospital will have these administered by a trained research associate visiting them at home or an agreed location. Patients will receive a follow up telephone call at six weeks to record any VTE events since surgery. OKS, EQ-5D and complications questionnaire will be posted to the patient at 6 and 12 months. We will use techniques common in long-term cohort studies to ensure minimum loss to follow-up, such as collection of next of kin contact addresses and telephone numbers, mobile telephone numbers and email addresses. A maximum of three attempts will be made acquire outcome data at each time point.

Sample size calculation

As this is a pilot trial and not designed to measure effect a formal sample size calculation is not required (the statistical analysis will be largely descriptive). We propose seeking to recruit and obtain primary clinical outcome data for 50 patients for descriptive analysis.

Statistical analysis

Standard descriptive statistics (e.g. medians and ranges or means and variances, dependent on the distribution of the outcome) and graphical plots showing correlations will be presented for the primary and secondary outcome measures. Baseline data will be summarised to check comparability between treatment arms, and to highlight any characteristic differences between those individuals in the trial, those ineligible, and those eligible but withholding consent. Exploratory analyses using regression techniques will be used to assess change in total volume of acute brain lesions in the two treatment groups and investigate the relationship between cognitive test scores and total volume of acute brain lesions. Linear regression will also be used to estimate the proportion of the variation in total volume of acute brain lesions that may be explained by change in cognition score between baseline and first post-operative assessment. To further investigate the extent to which cognition score is a surrogate for total volume of acute brain lesions we will use logistic regression to predict treatment group from change in the total volume of acute brain lesions. The relationship between cognitive symptoms and the MRI data will also be evaluated using voxel-based lesion-symptom mapping, where MRI intensity changes are associated to cognitive deficits using either classification scores (deficit or not) or continuous measures of cognition.

The routine statistical analysis will mainly be carried out using STATA (Data Analysis and Statistical Software).

Data Management

After baseline demographic data is collected, a unique trial number will identify patients. All data collected will be entered into a secure trial database held at Warwick Clinical Trials Unit (WCTU).

For peer review only - http://bmjopen.bmj.com/site/about/guidelines.xhtml

Identifiable patient information will be held in a locked filing cabinet and coded with a patient trial number. The WCTU quality assurance manager will audit trial records in accordance with Standard Operating Procedures. Outcomes will not be analysed until all primary outcome data is collected.

Trial oversight

The Trial Management Group, consisting of the staff involved in the day-to-day running of the study, will meet monthly. Significant issues arising from management meetings will be referred to the Trial Steering Committee as appropriate. The trial will be guided by a Trial Steering Committee (TSC), a group of respected and experienced personnel and trialists as well as lay representatives. The TSC will have an independent Chairperson. At least two formal TSC meeting will be held, one before the trial starts and one before recruitment to the trial completes. As this is a small feasibility study there will not be a data monitoring committee. Adverse events and SAE's will be monitored by the investigators. Adverse events will be assessed for causality within 24 hours of notification and patients followed up as per protocol. The trial may be stopped prematurely if mandated by ethics committee, a major unexpected safety concern arises or funding ceases. Any proposed changes to the protocol will first be reviewed by the TSC and if approved submitted to the trial sponsor, funding body and local research ethics committee. All approved protocols will be marked by a version number and date. Requests for access to the final dataset will be overseen by the TSC. Reasonable requests will then be given access to a full anonymised dataset.

Patient and Public Involvement

The study has a Patient and Public Involvement (PPI) group which includes two patients (CG and JS) who have previously undergone TKR surgery and have experience of the intervention being evaluated and its burden on patients. The study also has one Public Member (JD). The PPI group helped develop this study protocol, the associated patient information material and the outcome measures to be used through an ative participation in both TMG and TSC meetings. The PPI group critically evaluate study progress and are active study collaborators alongside other members of the research team. The PPI group have taken part in training events to help them participate fully as members of the study team. The PPI group will facilitate the preparation of information about the results of this study and any future planned larger scale study to inform participants of this study, pateints and the wider public. This information wil be disseminated through a mixture of social media, written information sheets and peer reviewed published papers.

B - Integrated qualitative research study

Method

Patients

For peer review only - http://bmjopen.bmj.com/site/about/guidelines.xhtml

In depth semi-structured interviews amongst with randomised patients and potential patients who decline to take part in the pilot trial will help understand people's views regarding participation in the pilot trial. A purposive sample of around 30 people (evenly split between recruited and not recruited) will be interviewed, based on patient demographics (including age, gender and socioeconomic status).[30,31] Patients will be recruited by a trained research associate and interviews will then be undertaken at a time convenient to the patient.

Surgeons

A survey will be undertaken amongst surgeons who routinely do TKR surgery. The survey will help gauge the extent to which this community is in clinical equipoise and would be willing to engage with, and support a larger trial. We will use a web-based survey of members of the British Association for Surgery of the Knee (BASK). BASK has a UK membership of over 100 practising knee surgeons of whom the majority routinely undertake TKR surgery. A sample of at least 20 BASK surgeons (both those clearly in equipoise and those that are not) will be invited to take part in more in depth qualitative interviews. We will use the general theory of implementation to investigate and understand the core constructs of sense making processes of patients and surgeons regarding the feasibility of a full trial and to help understand any barriers and enablers for a larger trial.

Digital audio recordings of interviews will be transcribed verbatim, checked and anonymised. Data will be managed and shared using N-Vivo analysis software. The analysis will be informed by a constant comparative approach, where early analysis informs subsequent data collection, and data analysis takes place alongside data collection.[30] This means insights from patients and surgeons can be explored further in the on-going interviews. Data is coded from the start of data collection, and new data is compared with existing data. Codes are compared, categories are constructed and explored to ensure they are robust and then they are linked with relevant theoretical literature. A technique of triangulation protocol will be used to facilitate the integration of the findings of the qualitative research with the quantitative data thereby helping to determine the overall feasibility of a full trial.[32] The data from patient interviews will also be used to help design optimal patient information materials for a larger trial.

C - Multi-centre cohort study

The National Joint Registry (NJR) is a population-based registry of joint replacements in the UK and covers both the NHS and private sectors. From April 2003 to December 2003 the NJR collected data on the use of tourniquets for TKR surgery in England and Wales. At this stage there were 406 hospitals listed within the NJR system (NHS hospitals, independent sector hospitals, and Treatment Centres - both NHS funded and privately funded) and of these, 384 returned data (i.e. 94%).The NJR dataset also contains many other key variables which are known to affect mortality, implant survivorship (revision rate) and the risk of VTE (1) use and type of VTE prophylaxis (such as low molecular weight heparin, aspirin, intermittent calf pump) (2) type of implant used (3) use of cement (4) basic patient

For peer review only - http://bmjopen.bmj.com/site/about/guidelines.xhtml

demographics including age, body mass index (BMI) and American Society of Anaesthesiologists (ASA) grade.

Method

We will access NJR data for the period in which tourniquet use was collected as part of the minimum dataset standard for knee replacement surgery – April 2003 to December 2003. We will analyse only data from primary cemented TKRs. Tourniquet use along with other routinely collected NJR baseline variables: age at time of surgery, sex, American Society of Anaesthesiologists (ASA) classification will be analysed to measure for independent effects on all cause revision. A tourniquet will be classed as used if even for a selective period of the procedure such as cementation. Sociodemographics, co-morbidities, VTEs, cerebrovascular accident (CVAs or stroke) and length of hospital stay will be obtained by using patient identifiers (NHS Number, gender, date of birth, postcode, procedure code and local patient identifier code) from the NJR to link the data to the Hospital Episode Statistics (HES) database. The HES data will provide details of VTE, socio-demographics, comorbidities and hospital length of stay for the TKR. The study will specifically estimate differences between groups (tourniquet or no tourniquet) for the following:

i. Baseline characteristics (age, BMI, ASA, co-morbidities, type of VTE prophylaxis, type of implant, socio-demographics)
ii. All cause mortality up to 12 months
iii. Length of inpatient hospital stay
iv. Risk of surgical complications up to 30 days
v. Risk of VTE up to 12 months
vi. Risk of CVA up to 12 months
vii. Risk of revision at 1, 5 and 10 years

Statistical Analysis

The Kaplan-Meier method will be used to calculate mortality and revision rates in those who underwent TKR with and without a tourniquet. Multivariate logistic regression will be used to assess the effect of age, body mass index, socio-economic status, tourniquet use, type of VTE prophylaxis, American Society of Anaesthesiologists (ASA) grade, co-morbidities and type of TKR implant (cemented or uncemented) on risk of VTE, CVA and risk of revision. The results of these analyses will inform the planning of a subsequent definitive full trial, if such a trial is feasible.

Conclusions - safety and feasibility evaluation and pathway to a full trial

A workshop will be held at the end of the feasibility study, involving key stakeholders (approximately 30 participants), patients, surgeons, researchers, allied healthcare professionals and healthcare policy makers. The workshop will have an independent chairperson. Using consensus conference methodology, delegates will be presented with results from the pilot RCT, retrospective multi-centre cohort study, existing published data and where applicable pooled data from all three sources. The purpose will be to agree a consensus statement on the appropriateness and feasibility of proceeding to a full trial and the most appropriate primary outcome measure for a full trial.

For peer review only - http://bmjopen.bmj.com/site/about/guidelines.xhtml

A full definitive trial will be deemed feasible if:

i. The pilot RCT and integrated qualitative study suggest patients can be recruited and surgeons are prepared to perform surgery with or without a tourniquet.

AND

ii. The pilot trial identifies either a measure of cognitive brain function that accurately reflects symptomatic brain emboli or an alternative primary/co-primary outcome measure is identified such as pain, symptomatic VTE, revision rates, OKS or EQ5D-5L scores that can be accurately and robustly collected in a full trial.

AND

iii. Key stakeholders (patients, surgeons, public representatives and researchers) agree at an end of study consensus conference that in light of the feasibility study and existing published research there remains insufficient data to make recommendations about the safety and clinical effectiveness of tourniquets and that a full trial is feasible and there is an appropriate primary / co-primary outcome measure for assessment.

Acknowledgments, Competing Interests, Funding and sponsorship

Acknowledgements

We would also like to acknowledge the support of Warwick Clinical Trials unit and University Hospital Coventry and Warwickshire.

The operating surgeons involved in this study include: A Ali, M Blakemore, K El-Bayouk, P Foguet, A Kotecha, R King, J McArthur, A Metcalfe, M Margetts, S Patil, B Riemer, K Sarantos, F Shah, T Spalding, P Thompson, R Westerman.

We would like to thank J Smith (Patient Member), C Goulden (Patient Member) and Jan Dixon (Public Member) who are members of the Patient Public Involvement group for working with the study team to collaborate on SAFE-TKR and the valuable contributions they have made to the design and success of the study to date.

Competing Interests:

All authors have no competing interests to declare.

Ethics and Dissemination

For peer review only - http://bmjopen.bmj.com/site/about/guidelines.xhtml

The study trial obtained approval from the National Research Ethics Committee (NRES) West Midlands – Edgbaston (15/WM/0455) on the 27th January 2016.

We aim to publish the results in at least one high impact peer reviewed journal. The results of the trial will also be disseminated via patient information material produced in collobroation with our Puplic Patient Involvement group. All key study findings will be presented at national and international conferences e.g. British Orthopaedic Association (BOA), British Association of Specialist Knee Surgeons (BASK) and America Academy of Orthopedic Surgeons (AAOS).

Funding and sponsorship

The study protocol represents research funded by a National Institute for Health Research (NIHR) Post-Doctoral Fellowship Award (PDF-2015-08-108). The study is jointly sponsored by the University of Warwick and University Hospitals Coventry & Warwickshire NHS Trust. The trial sponsors provide ultimate approval of all new versions of the protocol before they become live. Both the funders and sponsors are required to provide final approval before publication of any study material.

Author Contributions

PW: Chief investigator, study conception, study design, data collection, drafted and reviewed final manuscript

IA: Data collection, drafted and reviewed final manuscript

AM: Principal investigator, drafted and reviewed final manuscript

AP: Study design, drafted and reviewed final manuscript

KS: Study design, drafted and reviewed final manuscript

CH: Study design, data analysis, drafted and reviewed final manuscript

HP: Data analysis, drafted and reviewed final manuscript

JW: Data analysis, drafted and reviewed final manuscript

BR: Trial manager, data collection, drafted and reviewed final manuscript

JB: Trial manager, data collection, drafted and reviewed final manuscript

MU: Study design, drafted and reviewed final manuscript

SAFE-TKR Study Group: Study design and reviewed final manuscript

Collaborators

For peer review only - http://bmjopen.bmj.com/site/about/guidelines.xhtml

SAFE-TKR Study Group: B Rahman, J Brown, C Goulden, K Seers, A Metcalfe, J Smith, J Dixon, J Warwick, H Parsons, N Demeyere, C Hutchinson, I Ahmed, A Price, M Underwood, P Wall.

Appendices

Appendix 1 – Pilot RCT Patient Information Sheet

See supplementary file 1

Appendix 2 – Pilot RCT Patient Consent Form

See supplementary file 2

References

1. Callahan CM, Drake BG, Heck DA, Dittus RS. Patient outcomes following tricompartmental total knee replacement. A meta-analysis. *Jama 1994;271-17:1349-57.*
2. Zhang W, Li N, Chen S, Tan Y, Al-Aidaros M, Chen L. The effects of a tourniquet used in total knee arthroplasty: a meta-analysis. *J Orthop Surg Res 2014;9-1:13.*
3. National Joint Registry for England and Wales. National Joint Registry for England and Wales 1st Annual Report 2004.
4. Parvizi J, Diaz-Ledezma C. Total knee replacement with the use of a tourniquet: more pros than cons. *Bone Joint J 2013;95-B-11 Suppl A:133-4.*
5. Tai TW, Lin CJ, Jou IM, Chang CW, Lai KA, Yang CY. Tourniquet use in total knee arthroplasty: a meta-analysis. *Knee Surg Sports Traumatol Arthrosc 2011;19-7:1121-30.*
6. Smith TO, Hing CB. Is a tourniquet beneficial in total knee replacement surgery? A meta-analysis and systematic review. *Knee 2010;17-2:141-7.*
7. Alcelik I, Pollock RD, Sukeik M, Bettany-Saltikov J, Armstrong PM, Fismer P. A comparison of outcomes with and without a tourniquet in total knee arthroplasty: a systematic review and meta-analysis of randomized controlled trials. *J Arthroplasty 2012;27-3:331-40.*
8. Berman AT, Parmet JL, Harding SP, Israelite CL, Chandrasekaran K, Horrow JC, Singer R, Rosenberg H. Emboli observed with use of transesophageal echocardiography immediately after tourniquet release during total knee arthroplasty with cement. *J Bone Joint Surg Am 1998;80-3:389-96.*
9. Sulek CA, Davies LK, Enneking FK, Gearen PA, Lobato EB. Cerebral microembolism diagnosed by transcranial Doppler during total knee arthroplasty: correlation with transesophageal echocardiography. *Anesthesiology 1999;91-3:672-6.*
10. Deo H, West G, Butcher C, Lewis P. The prevalence of cognitive dysfunction after conventional and computer-assisted total knee replacement. *Knee 2011;18-2:117-20.*
11. Chan AW, Tetzlaff JM, Altman DG, Laupacis A, Gotzsche PC, Krleza-Jeric K, Hrobjartsson A, Mann H, Dickersin K, Berlin JA, Dore CJ, Parulekar WR, Summerskill WS, Groves T, Schulz KF, Sox HC, Rockhold FW, Rennie D, Moher D. SPIRIT 2013 statement: defining standard protocol items for clinical trials. *Ann Intern Med 2013;158-3:200-7.*
12. Schulz KF, Altman DG, Moher D, Group C. CONSORT 2010 statement: updated guidelines for reporting parallel group randomised trials. *Int J Surg 2011;9-8:672-7.*
13. Altman DG, Bland JM. Treatment allocation by minimisation. *BMJ 2005;330-7495:843.*

For peer review only - http://bmjopen.bmj.com/site/about/guidelines.xhtml

14. Fairbairn TA, Mather AN, Bijsterveld P, Worthy G, Currie S, Goddard AJ, Blackman DJ, Plein S, Greenwood JP. Diffusion-weighted MRI determined cerebral embolic infarction following transcatheter aortic valve implantation: assessment of predictive risk factors and the relationship to subsequent health status. *Heart 2012;98-1:18-23.*
15. Ghanem A, Muller A, Nahle CP, Kocurek J, Werner N, Hammerstingl C, Schild HH, Schwab JO, Mellert F, Fimmers R, Nickenig G, Thomas D. Risk and fate of cerebral embolism after transfemoral aortic valve implantation: a prospective pilot study with diffusion-weighted magnetic resonance imaging. *J Am Coll Cardiol 2010;55-14:1427-32.*
16. Gress DR. The problem with asymptomatic cerebral embolic complications in vascular procedures: what if they are not asymptomatic? *J Am Coll Cardiol 2012;60-17:1614-6.*
17. Bijuklic K, Wandler A, Tubler T, Schofer J. Impact of asymptomatic cerebral lesions in diffusion-weighted magnetic resonance imaging after carotid artery stenting. *JACC Cardiovasc Interv 2013;6-4:394-8.*
18. Blum S, Luchsinger JA, Manly JJ, Schupf N, Stern Y, Brown TR, DeCarli C, Small SA, Mayeux R, Brickman AM. Memory after silent stroke: hippocampus and infarcts both matter. *Neurology 2012;78-1:38-46.*
19. Arvanitakis Z, Leurgans SE, Barnes LL, Bennett DA, Schneider JA. Microinfarct pathology, dementia, and cognitive systems. *Stroke 2011;42-3:722-7.*
20. Karoutas G, Milonas I, Artemis N, Karacostas D, Pitas J, Logothetis J. The pharmacological effects of buflomedil in patients with multi-cerebral infarcts dementia: an open, preliminary assessment. *Curr Med Res Opin 1987;10-6:380-9.*
21. Stolwyk RJ, O'Neill MH, McKay AJ, Wong DK. Are cognitive screening tools sensitive and specific enough for use after stroke? A systematic literature review. *Stroke 2014;45-10:3129-34.*
22. Demeyere N, Slavkova E, Riddoch MJ, Humphreys GW. The Oxford Cognitive Screen (OCS): Validation of a stroke-specific short cognitive screening tool. *Psychological Assessment 2015 (in press).*
23. Petersen RC, Smith GE, Waring SC, Ivnik RJ, Tangalos EG, Kokmen E. Mild cognitive impairment: clinical characterization and outcome. *Arch Neurol 1999;56-3:303-8.*
24. Saczynski JS, Marcantonio ER, Quach L, Fong TG, Gross A, Inouye SK, Jones RN. Cognitive trajectories after postoperative delirium. *N Engl J Med 2012;367-1:30-9.*
25. Folstein MF, Folstein SE, McHugh PR. "Mini-mental state". A practical method for grading the cognitive state of patients for the clinician. *J Psychiatr Res 1975;12-3:189-98.*
26. Marques EM, Jones HE, Elvers KT, Pyke M, Blom AW, Beswick AD. Local anaesthetic infiltration for peri-operative pain control in total hip and knee replacement: systematic review and meta-analyses of short- and long-term effectiveness. *BMC Musculoskelet Disord 2014;15:220.*
27. Dawson J, Fitzpatrick R, Murray D, Carr A. Questionnaire on the perceptions of patients about total knee replacement. *J Bone Joint Surg Br 1998;80-1:63-9.*
28. EuroQol G. EuroQol--a new facility for the measurement of health-related quality of life. *Health Policy 1990;16-3:199-208.*
29. Walters SJ, Brazier JE. Comparison of the minimally important difference for two health state utility measures: EQ-5D and SF-6D. *Qual Life Res 2005;14-6:1523-32.*
30. Charmaz KC. *Constructing grounded theory: A practical guide through qualitative analysis.* 2006.
31. Marshall B, Cardon P, Poddar A, Fontenot R. DOES SAMPLE SIZE MATTER IN QUALITATIVE RESEARCH?: A REVIEW OF QUALITATIVE INTERVIEWS IN IS RESEARCH. *Journal of Computer Information Systems 2013.*
32. O'Cathain A, Murphy E, Nicholl J. Three techniques for integrating data in mixed methods studies. *BMJ 2010;341:c4587.*

For peer review only

For peer review only - http://bmjopen.bmj.com/site/about/guidelines.xhtml

WARWICK
THE UNIVERSITY OF WARWICK

University Hospitals Coventry and Warwickshire NHS Trust

Safety and Feasibility Evaluation of Tourniquets For Total Knee Replacement (SAFE-TKR) Study

Patient Information Sheet

Pilot Randomised Controlled Trial
Chief Investigator: Peter Wall

You are invited to take part in our research study. Before you decide whether to take part we would like you to understand why the research is being done and what it would involve for you. Once you have had a chance to read and understand this information sheet a member of our team will go through the information with you and answer any questions you may have.

Background

When you have a total knee replacement, most surgeons apply a tourniquet around the thigh before starting the operation. The tourniquet is a device wrapped around your thigh which when inflated squeezes the thigh, to reduce the blood flow, helping to create a bloodless operative field. Excessive blood within the operative field can interfere with visualising the knee joint during surgery and so a tourniquet is often helpful but by no means essential. There is some limited evidence that a bloodless field during surgery helps to improve the fixation of the knee replacement implant to the bone which could help the implant to last longer.

However, there is some evidence that suggests using a tourniquet during knee replacement surgery may have some risks. Potentially, these include an increased risk of developing a blood clot (so called Deep Vein Thrombosis and Pulmonary Embolism) after surgery. There is also a theoretical risk that when the tourniquet is deflated at the end of the operation that small blood clots that have accumulated in the leg could travel to the brain and cause damage. In addition using a tourniquet during surgery may lead to increased swelling and pain after the operation.

The aim of this study is to help identify and assess all these benefits and risks. By monitoring you closely before, during and after surgery and performing detailed brain scans (Magnetic Resonance Imaging - MRI) before and the day after your operation we will try to establish if there is any evidence that using a tourniquet increases your risk of a blood clot traveling to the brain.

It is important to understand that most patients currently have knee replacement surgery performed using a tourniquet and it remains a very safe procedure. This study will however help to make it even safer and determine for future patients the most effective and safe way of performing a knee replacement.

If you have any questions please contact the Study Manager, Ms Bushra Rahman by either telephoning 02476 968626 / 0787 6876 978 or emailing safe-tkr@warwick.ac.uk
For more information about the study visit:
http://www2.warwick.ac.uk/fac/med/research/hscience/ctu/trials/safetkr

For peer review only - http://bmjopen.bmj.com/site/about/guidelines.xhtml

Why have you been invited?
We are asking up to 50 patients who are going to have a total knee replacement to take part in the study. However, if you have a pacemaker, metallic clips in your brain or are claustrophobic you are not eligible to take part because the study involves the use of the MRI scanner.

Do I have to take part?
It is up to you to decide. If you decide to take part we will ask you to sign a consent form. You are free to withdraw at any time without giving a reason. This will not affect the care you receive.

What will happen to me if I take part?
If you decide to take part you will be asked to sign a consent form. You will then be invited to one of the two treatments. In order to make our study work it is crucial that we have equal numbers of volunteers in each treatment group and that the one you (are invited to) join is determined by a sophisticated machine designed for this purpose, not influenced by us. More information about the two possible treatments is given below. Whichever treatment you have your care will be based on meeting your individual needs, and you will continue with the same team of surgeons and healthcare professionals. To ensure the study is as robust as possible and prevent any bias we will inform you whether your surgery involved a tourniquet or not at the end of the study period i.e. 12 months from when you joined the study.

Before the Surgery
We will ask you to complete a questionnaire and a trained member of the research team will ask you some questions. We will also arrange for you to have a MRI brain scan before your operation. The scan will take 20 minutes to complete and is non-invasive. The scan does not use radiation; instead it uses a magnetic field to look at structures in the brain. The MRI scan would involve positioning yourself on a bed, whilst going through a "doughnut shaped" scanner (see image below). During the scan you will be asked to keep still. The scanner can be noisy and headphones will be provided. There will be a radiographer assisting and talking to you at all times throughout the process.

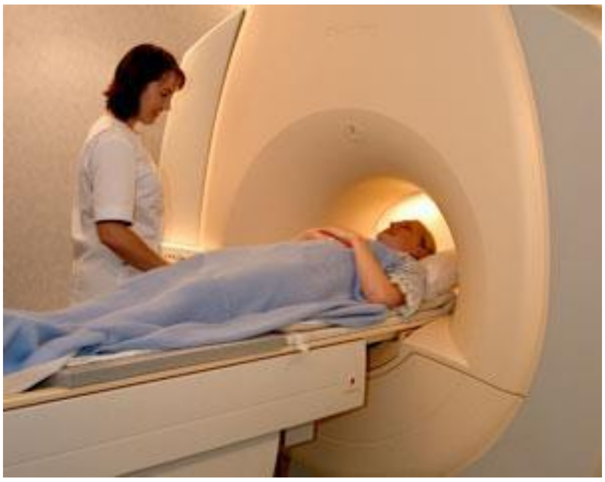
Image of a patient having an MRI Scan

If you have any questions please contact the Study Manager, Ms Bushra Rahman by either telephoning 02476 968626 / 0787 6876 978 or emailing safe-tkr@warwick.ac.uk
For more information about the study visit:
http://www2.warwick.ac.uk/fac/med/research/hscience/ctu/trials/safetkr

For peer review only - http://bmjopen.bmj.com/site/about/guidelines.xhtml

WARWICK
THE UNIVERSITY OF WARWICK

University Hospitals NHS
Coventry and Warwickshire
NHS Trust

After the Surgery

Following the operation you will have another MRI brain scan, before you are discharged from hospital. In the days after your operation you will also be asked to answer some questions to help us test your memory and other brain functions as well as questions about any thigh pain you may be experiencing and how you are feeling in general. These questions will be undertaken by a trained member of the research team at Day 1 and 2 and 7 this may take upto 40 minutes. We will arrange a visit if you are already discharged after your operation. We will call you by telephone 6 weeks after your operation to find out about any problems after the surgery. We will also ask you to complete a postal questionnaire at 6 months and 12 months after your operation to find out how your knee replacement is doing. If you agree we can send you a text message or email near the time to remind you that the questionnaire is due. If you need help completing a questionnaire, a researcher can contact you by phone to help you complete it.

Finally to help understand the reasons why you decided to, or declined to take part in the research we may invite you to undertake a short interview with a trained researcher. This interview will help us to improve the research experience for future patients.

Which treatments are you comparing?

The two treatments that are being compared are:

- *Total knee replacement surgery using a tourniquet*
- *Total knee replacement surgery not using a tourniquet*

What are the possible benefit and risks of taking part?

There are no specific benefits to taking part. Both treatments are designed to help you and your knee arthritis. Your surgeon is able to do the total knee replacement either with or without a tourniquet but it is not clear which way is better. There are some small risks associated with both techniques including blood clots, infection and pain.

What if new information becomes available?

Sometimes during the course of a study, new information becomes available about the treatments that are being studied. If this happens, someone from our research team will tell you about it and discuss with you whether you want to continue in the study. If you decide to withdraw, you can discuss your continued care with your doctor. If you decide to continue in the study you might be asked to sign an updated consent form. Also, on receiving new information, we might consider it to be in your best interests to withdraw you from the study. If this happens we will explain the reasons to you and arrange for your care to continue.

What happens when the research study stops?

You will participate in the study for one year. If you are having any problems relating to the knee surgery after this time, we will arrange for you to see your specialist to continue your care.

If you have any questions please contact the Study Manager, Ms Bushra Rahman by either telephoning 02476 968626 / 0787 6876 978 or emailing safe-tkr@warwick.ac.uk
For more information about the study visit:
http://www2.warwick.ac.uk/fac/med/research/hscience/ctu/trials/safetkr

For peer review only - http://bmjopen.bmj.com/site/about/guidelines.xhtml

WARWICK
THE UNIVERSITY OF WARWICK

University Hospitals Coventry and Warwickshire NHS Trust

What if something goes wrong?
In the event that something goes wrong and you are harmed during the research due to someone's negligence, then you may have grounds for legal action for compensation against the University of Warwick (contact the Head of Research Governance,

Research Impact Services, University House, University of Warwick, Coventry, CV4 8UW or by email researchgovernance@warwick.ac.uk or telephone 02476 522746)

Will my taking part in this study be kept confidential?
All information which is collected about you during the course of the research will be kept strictly confidential. Research data including your name and address will be sent to the University of Warwick so that research staff can stay in touch with you over the course of the year, and send you follow-up questionnaires at 6 and 12 months by post or email. These details will be sent from the hospital by secure means, and kept in locked filing cabinets or in password-protected computer databases accessible only to essential research personnel at the University of Warwick. All other information about you which leaves the hospital will have your name and address removed so that you cannot be recognised from it. If you agree, your GP and other doctors who may treat you, but are not part of this study, will be notified that you are taking part in this study.

What will happen to the results of the research study?
At the end of the study we will publish the findings in medical journals and at medical conferences. You will not be identified in any reports or publications resulting from the study. If you would like to obtain a copy of the published results, please email safe-tkr@warwick.ac.uk.

Who has reviewed this project?
This study has been reviewed and approved by West Midlands – Edgbaston Research Ethics Committee REC Ref. 15/WM/0455. Approval was granted on 27th January 2016

Who is organising and funding the research?
This research has been organised by the University of Warwick and funded by the National Institute for Health Research, UK.

Contacts for further information
If, at any time, you would like further information about this research study you may contact the study manager (Ms Bushra Rahman) by either telephoning 02476 968626 / or emailing safe-tkr@warwick.ac.uk.

For independent advice contact the PALS service (Patient Advice Liaison Service) www.pals.nhs.uk / 0800 0284203.

Thank you for considering participation in this study and for taking time to read this information sheet

If you have any questions please contact the Study Manager, Ms Bushra Rahman by either telephoning 02476 968626 / 0787 6876 978 or emailing safe-tkr@warwick.ac.uk
For more information about the study visit:
http://www2.warwick.ac.uk/fac/med/research/hscience/ctu/trials/safetkr

For peer review only - http://bmjopen.bmj.com/site/about/guidelines.xhtml

WARWICK
THE UNIVERSITY OF WARWICK

University Hospitals Coventry and Warwickshire NHS Trust

Office Use Only:
Participant ID: ☐☐☐

CONSENT FORM

Pilot Randomised Controlled Trial

SAFE-TKR Study

Chief Investigator: Mr Peter Wall

(Please initial)

1. I confirm that I have read and understand the information sheet dated 09th August 2017 Version 4 for the above study. I have had the opportunity to consider the information, ask questions and have had these answered satisfactorily. ☐

2. I understand that my participation is voluntary and that I am free to withdraw at any time, without giving any reason, without my medical care or legal rights being affected. ☐

3. I understand that relevant sections of any of my medical notes and data collected during the study may be looked at by responsible individuals from the University of Warwick, from regulatory authorities, or from the NHS trust, where it is relevant to my taking part in this research. I give permission for these individuals to have access to my records. ☐

4. I agree to my GP being informed of my participation ☐

5. I agree to being sent a text message reminding that my follow up questionnaire is due. ☐

6. I agree to take part in the above study. ☐

Name of Patient	Signature	Date
Name of Person taking consent	Signature	Date

FOR OFFICE USE ONLY

Copies: ☐ 1 for Patient ☐ 1 for Hospital notes ☐ Original document retained in site file

Signature ____________________ Date ____________

For peer review only

For peer review only - http://bmjopen.bmj.com/site/about/guidelines.xhtml

STANDARD PROTOCOL ITEMS: RECOMMENDATIONS FOR INTERVENTIONAL TRIALS

SPIRIT 2013 Checklist: Recommended items to address in a clinical trial protocol and related documents*

Section/item	Item No	Description	Addressed on page number
**Administrative information**			
Title	1	Descriptive title identifying the study design, population, interventions, and, if applicable, trial acronym	_____1________
Trial registration	2a	Trial identifier and registry name. If not yet registered, name of intended registry	_____2________
	2b	All items from the World Health Organization Trial Registration Data Set	1-11 & trial registration page
Protocol version	3	Date and version identifier	_____4________
Funding	4	Sources and types of financial, material, and other support	_____2 & 10
Roles and responsibilities	5a	Names, affiliations, and roles of protocol contributors	_______11_
	5b	Name and contact information for the trial sponsor	_______11_______
	5c	Role of study sponsor and funders, if any, in study design; collection, management, analysis, and interpretation of data; writing of the report; and the decision to submit the report for publication, including whether they will have ultimate authority over any of these activities	________10______
	5d	Composition, roles, and responsibilities of the coordinating centre, steering committee, endpoint adjudication committee, data management team, and other individuals or groups overseeing the trial, if applicable (see Item 21a for data monitoring committee)	______8 & 10___

For peer review only - http://bmjopen.bmj.com/site/about/guidelines.xhtml

Introduction

Background and rationale	6a	Description of research question and justification for undertaking the trial, including summary of relevant studies (published and unpublished) examining benefits and harms for each intervention	______3/4_____
	6b	Explanation for choice of comparators	________3/4_____
Objectives	7	Specific objectives or hypotheses	_____3/4_______ _
Trial design	8	Description of trial design including type of trial (eg, parallel group, crossover, factorial, single group), allocation ratio, and framework (eg, superiority, equivalence, noninferiority, exploratory)	____4-7__

Methods: Participants, interventions, and outcomes

Study setting	9	Description of study settings (eg, community clinic, academic hospital) and list of countries where data will be collected. Reference to where list of study sites can be obtained	_____4________
Eligibility criteria	10	Inclusion and exclusion criteria for participants. If applicable, eligibility criteria for study centres and individuals who will perform the interventions (eg, surgeons, psychotherapists)	_____4________
Interventions	11a	Interventions for each group with sufficient detail to allow replication, including how and when they will be administered	_____5________
	11b	Criteria for discontinuing or modifying allocated interventions for a given trial participant (eg, drug dose change in response to harms, participant request, or improving/worsening disease)	_____5________
	11c	Strategies to improve adherence to intervention protocols, and any procedures for monitoring adherence (eg, drug tablet return, laboratory tests)	_____7/8_______
	11d	Relevant concomitant care and interventions that are permitted or prohibited during the trial	_____5________
Outcomes	12	Primary, secondary, and other outcomes, including the specific measurement variable (eg, systolic blood pressure), analysis metric (eg, change from baseline, final value, time to event), method of aggregation (eg, median, proportion), and time point for each outcome. Explanation of the clinical relevance of chosen efficacy and harm outcomes is strongly recommended	_____5/6_______

For peer review only

Participant timeline	13	Time schedule of enrolment, interventions (including any run-ins and washouts), assessments, and visits for participants. A schematic diagram is highly recommended (see Figure)	_____6/7______
Sample size	14	Estimated number of participants needed to achieve study objectives and how it was determined, including clinical and statistical assumptions supporting any sample size calculations	_____7_______
Recruitment	15	Strategies for achieving adequate participant enrolment to reach target sample size	_____7______

Methods: Assignment of interventions (for controlled trials)

Allocation:

Sequence generation	16a	Method of generating the allocation sequence (eg, computer-generated random numbers), and list of any factors for stratification. To reduce predictability of a random sequence, details of any planned restriction (eg, blocking) should be provided in a separate document that is unavailable to those who enrol participants or assign interventions	_______4/5_____
Allocation concealment mechanism	16b	Mechanism of implementing the allocation sequence (eg, central telephone; sequentially numbered, opaque, sealed envelopes), describing any steps to conceal the sequence until interventions are assigned	_____4/5______
Implementation	16c	Who will generate the allocation sequence, who will enrol participants, and who will assign participants to interventions	_____4/5______
Blinding (masking)	17a	Who will be blinded after assignment to interventions (eg, trial participants, care providers, outcome assessors, data analysts), and how	______7_______
	17b	If blinded, circumstances under which unblinding is permissible, and procedure for revealing a participant's allocated intervention during the trial	______7_______

Methods: Data collection, management, and analysis

Data collection methods	18a	Plans for assessment and collection of outcome, baseline, and other trial data, including any related processes to promote data quality (eg, duplicate measurements, training of assessors) and a description of study instruments (eg, questionnaires, laboratory tests) along with their reliability and validity, if known. Reference to where data collection forms can be found, if not in the protocol	_____5-8______

For peer review only - http://bmjopen.bmj.com/site/about/guidelines.xhtml

	18b	Plans to promote participant retention and complete follow-up, including list of any outcome data to be collected for participants who discontinue or deviate from intervention protocols	_____7/8______
Data management	19	Plans for data entry, coding, security, and storage, including any related processes to promote data quality (eg, double data entry; range checks for data values). Reference to where details of data management procedures can be found, if not in the protocol	______8______
Statistical methods	20a	Statistical methods for analysing primary and secondary outcomes. Reference to where other details of the statistical analysis plan can be found, if not in the protocol	________7______
	20b	Methods for any additional analyses (eg, subgroup and adjusted analyses)	______7______
	20c	Definition of analysis population relating to protocol non-adherence (eg, as randomised analysis), and any statistical methods to handle missing data (eg, multiple imputation)	________7______
**Methods: Monitoring**			
Data monitoring	21a	Composition of data monitoring committee (DMC); summary of its role and reporting structure; statement of whether it is independent from the sponsor and competing interests; and reference to where further details about its charter can be found, if not in the protocol. Alternatively, an explanation of why a DMC is not needed	_____4/8_______
	21b	Description of any interim analyses and stopping guidelines, including who will have access to these interim results and make the final decision to terminate the trial	______4/8______
Harms	22	Plans for collecting, assessing, reporting, and managing solicited and spontaneously reported adverse events and other unintended effects of trial interventions or trial conduct	______8_______
Auditing	23	Frequency and procedures for auditing trial conduct, if any, and whether the process will be independent from investigators and the sponsor	______8_______
**Ethics and dissemination**			
Research ethics approval	24	Plans for seeking research ethics committee/institutional review board (REC/IRB) approval	________4______

For peer review only - http://bmjopen.bmj.com/site/about/guidelines.xhtml

Protocol amendments	25	Plans for communicating important protocol modifications (eg, changes to eligibility criteria, outcomes, analyses) to relevant parties (eg, investigators, REC/IRBs, trial participants, trial registries, journals, regulators)	_____8_________
Consent or assent	26a	Who will obtain informed consent or assent from potential trial participants or authorised surrogates, and how (see Item 32)	_____4/5________
	26b	Additional consent provisions for collection and use of participant data and biological specimens in ancillary studies, if applicable	_____4/5________
Confidentiality	27	How personal information about potential and enrolled participants will be collected, shared, and maintained in order to protect confidentiality before, during, and after the trial	______8_______
Declaration of interests	28	Financial and other competing interests for principal investigators for the overall trial and each study site	______10_______
Access to data	29	Statement of who will have access to the final trial dataset, and disclosure of contractual agreements that limit such access for investigators	______8_______
Ancillary and post-trial care	30	Provisions, if any, for ancillary and post-trial care, and for compensation to those who suffer harm from trial participation	______8_______
Dissemination policy	31a	Plans for investigators and sponsor to communicate trial results to participants, healthcare professionals, the public, and other relevant groups (eg, via publication, reporting in results databases, or other data sharing arrangements), including any publication restrictions	______10_______
	31b	Authorship eligibility guidelines and any intended use of professional writers	______10_______
	31c	Plans, if any, for granting public access to the full protocol, participant-level dataset, and statistical code	________8_____
**Appendices**			
Informed consent materials	32	Model consent form and other related documentation given to participants and authorised surrogates	___appendix 1+2 - ________Suppl file 1+2_
Biological specimens	33	Plans for collection, laboratory evaluation, and storage of biological specimens for genetic or molecular analysis in the current trial and for future use in ancillary studies, if applicable	______n/a______

For peer review only - http://bmjopen.bmj.com/site/about/guidelines.xhtml

For peer review only

*It is strongly recommended that this checklist be read in conjunction with the SPIRIT 2013 Explanation & Elaboration for important clarification on the items. Amendments to the protocol should be tracked and dated. The SPIRIT checklist is copyrighted by the SPIRIT Group under the Creative Commons “Attribution-NonCommercial-NoDerivs 3.0 Unported” license.

For peer review only

For peer review only - http://bmjopen.bmj.com/site/about/guidelines.xhtml
