# Supplementary material for: Safety and feasibility evaluation of tourniquets for total knee replacement (SAFE-TKR): study protocol
Source: BMJ Open. 2018 Apr 10;8(4):e022067. doi: 10.1136/bmjopen-2018-022067 (PMC5900804; doi:10.1136/bmjopen-2018-022067)
Supplement: Reviewer comments [file bmjopen-2018-022067.reviewer_comments.pdf]

PEER REVIEW HISTORY

BMJ Open publishes all reviews undertaken for accepted manuscripts. Reviewers are asked to complete a checklist review form (**http://bmjopen.bmj.com/site/about/resources/checklist.pdf**) and are provided with free text boxes to elaborate on their assessment. These free text comments are reproduced below.

ARTICLE DETAILS

**TITLE (PROVISIONAL)**	Safety and feasibility evaluation of tourniquets for total knee replacement (SAFE- TKR): study protocol
**AUTHORS**	Wall, Peter; Ahmed, Imran; Metcalfe, Andrew; Price, Andrew; Seers, Kate; Hutchinson, Charles; Parsons, Helen; Warwick, Jane; Rahman, Bushra; Brown, Jaclyn; Underwood, Martin; SAFE-TKR study group, On behalf of

This article was not externally reviewed at BMJ Open. Protocols that have been independently assessed prior to submission to BMJ Open are usually fast-tracked to publication on the grounds that further substantial changes will not be possible. This independent assessment will usually be external, independent review for both a substantial grant award from a non-commercial or government funder and ethics approval.
